# Causal Drift, Robust Signaling, and Complex Disease

**DOI:** 10.1371/journal.pone.0118413

**Published:** 2015-03-16

**Authors:** Andreas Wagner

**Affiliations:** 1 University of Zurich, Institute for Evolutionary Biology and Environmental Studies, Winterthurerstrasse 190, CH-8057 Zurich, Switzerland; 2 The Swiss Institute of Bioinformatics, Lausanne, Switzerland; 3 The Santa Fe Institute, Santa Fe, New Mexico; CRG, SPAIN

## Abstract

The phenotype of many regulatory circuits in which mutations can cause complex, polygenic diseases is to some extent robust to DNA mutations that affect circuit components. Here I demonstrate how such mutational robustness can prevent the discovery of genetic disease determinants. To make my case, I use a mathematical model of the insulin signaling pathway implicated in type 2 diabetes, whose signaling output is governed by 15 genetically determined parameters. Using multiple complementary measures of a parameter’s importance for this phenotype, I show that any one disease determinant that is crucial in one genetic background will be virtually irrelevant in other backgrounds. In an evolving population that drifts through the parameter space of this or other robust circuits through DNA mutations, the genetic changes that can cause disease will vary randomly over time. I call this phenomenon causal drift. It means that mutations causing disease in one (human or non-human) population may have no effect in another population, and vice versa. Causal drift casts doubt on our ability to infer the molecular mechanisms of complex diseases from non-human model organisms.

## Introduction

Complex genetic diseases like type 2 diabetes, Crohn’s disease, and schizophrenia are influenced by multiple genes whose identification is a great challenge for genomics [1–5]. Aside from environmental influences, complex gene interactions that affect disease risk are among the biggest obstacles to understand the causes of such complex diseases [6–11]. For example, genome-wide association studies of healthy and diseased individuals can often explain only little disease risk when they add the effect of many single nucleotide changes observed in diseased individuals [1, 5–7]. The causes of this phenomenon, which is also known as the ‘missing heritability’ problem, include the influence of rare mutations, many variants with weak effects, gene copy number changes, epigenetic changes, parent-of-origin effects, but especially non-additive or epistatic interactions among genetic polymorphisms at different loci [6, 8–13].

Genome-wide association studies can provide *statistical* evidence for a gene’s role in disease, but they do not resolve the *mechanistic* causes of disease. Progress in understanding these causes usually involves the analysis of regulatory or signaling circuits, in which molecular interactions among circuit components have been characterized. Examples include the potential role of insulin signaling in type II diabetes, or that of the mitogen activated protein kinase (MAPK) signaling circuit in hypertension [14–17]. Such circuits are complex and their analysis requires quantitative mathematical models. Most such models represent a circuit’s dynamics through ordinary differential equations whose state variables describe how the concentration or activity of circuit molecules, especially proteins, changes over time through interactions with other molecules. The strengths of these interactions are genetically encoded, for example in the amino acid sequences of protein interaction partners, and encapsulated in one or more model parameters. A circuit operating inside a cell usually receives an external signal, such as an insulin pulse in response to ingested sugar, and its response regulates downstream molecules such as transporters that can import glucose [14, 15]. Genetic change can alter the parameters that influence a circuit’s behavior, and in doing so, can change the circuit’s phenotype in a manner that leads to disease.

Understanding the phenotype of any one complex circuit is hindered by the usually unknown (and difficult to measure) values of most of its parameters, which may number in the dozens to hundreds [18]. The time-honored strategy of fitting the parameters to experimental phenotypic data has a serious limitation: Myriad sets of parameters can usually reproduce the same experimentally measured phenotype. Recent work therefore increasingly focuses on characterizing a model’s entire parameter space. This is done by sampling the usually high-dimensional space, allowing each parameter to vary over some biologically sensible range. The subset of *viable* parameters, i.e., parameter sets yielding a given phenotype, can have many dimensions and a complex geometry [19–28].

Because a circuit’s parameters are genetically determined, the size of the viable parameter set also reflects the amount of genetic change a circuit can tolerate while preserving its phenotype, that is, the circuit’s robustness to mutations [19–21, 29, 30]. Such robustness is a property of many biological systems [31]. But while circuits are to some extent robust, they are not equally robust to all genetic perturbations. Their phenotypes are much more sensitive to changes in some parameters than in others, a phenomenon that has been called ‘sloppy control’ [22, 32–35]. Especially important parameters could thus in principle serve as ‘choke points’ to help steer a circuit from a diseased to a normal state as part of a therapeutic intervention [36].

I here show that mutational robustness can impair our ability to detect genetic disease determinants. This is trivially true for systems with sloppy control: Genetically encoded parameters that do not affect a phenotype when altered through DNA mutations will be invisible in a genome-wide association study. But in genetically heterogenous populations the problem can be much worse. To make my case I use the insulin signaling circuit as a representative of many other, similarly robust circuits [22, 32]. The insulin circuit has been implicated in type II diabetes, a complex genetic disease and serious public health risk [14, 37, 38]. Disease manifestations include cellular resistance to pancreas- produced insulin, which reduces the rate at which cells import glucose and thus remove glucose from the bloodstream. The resulting hyperglycemia and has debilitating long-term effects such as blindness. The insulin signaling circuit, a small part of a highly complex glucose homeostasis system [38], responds to the presence of insulin at the cell surface and triggers the uptake of glucose into muscle cells and adipocytes. Naturally occurring mutations in circuit genes affect insulin resistance and diabetes risk [39–44]. Starting from a tractable yet experimentally validated mathematical model of this robust circuit [15], I sample its high-dimensional parameter space and study the effects of individual disease determinants (parameters) on its glucose uptake phenotype. Doing so for many different genetic backgrounds (different viable parameter sets) reveals that any one disease determinant that is crucial in one background will be modestly important in another and virtually irrelevant in yet another background. In an evolving population that explores the parameter space of such a circuit through DNA mutations, genetic determinants of disease can vary randomly over time. I call this phenomenon *causal drift*.

## Methods

**Fig 1:**
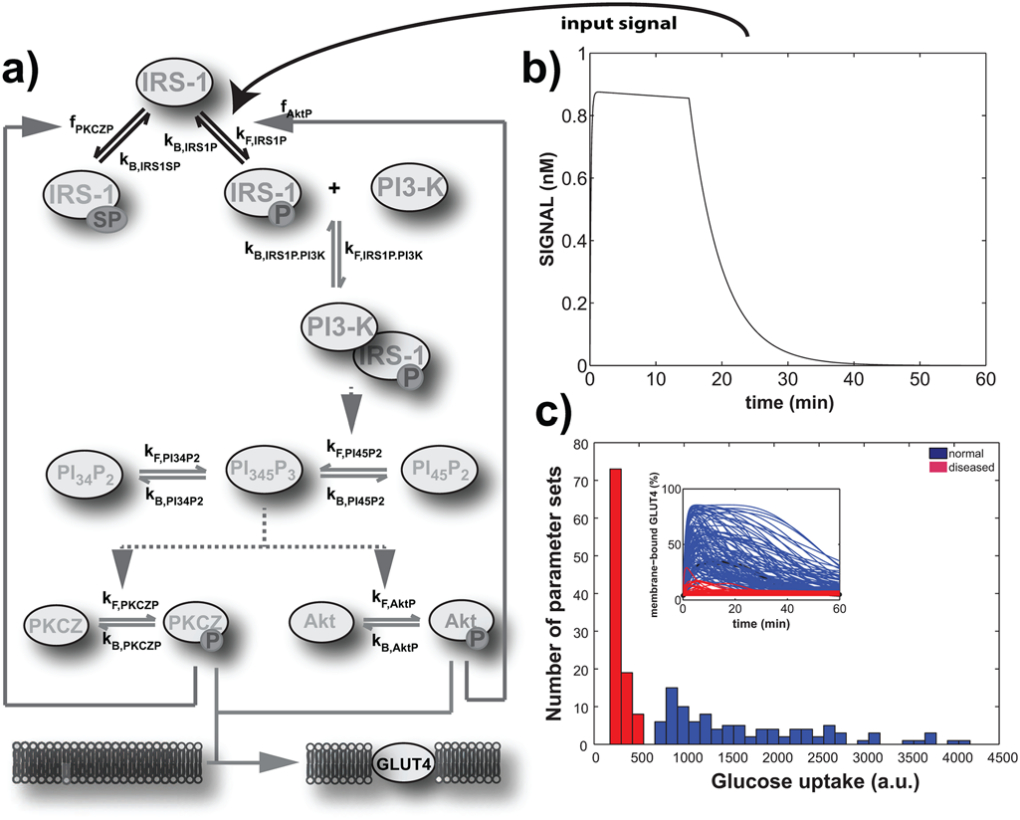
Insulin signaling model, input and output. **a)** Molecular interactions in the signaling pathway modeled here. Briefly, extracellular insulin leads to phosphorylation of the insulin receptor, which promotes the phosphorylation of *IRS*1 to yield *IRS*1*P*. The latter molecule associates with *PI*3*K* in a complex that triggers production of the second messenger *PI*_345_ *P*_3_, which activates the protein kinases *Akt* and *PKCZ*. These kinases then promote the translocation of the glucose transporter *GLUT*4 to the membrane, where it helps import glucose into the cell. Mass-action parameters that determine the rates of the respective reactions are indicated by a ‘**k**’ followed by a subscript. Activated *PKCZ* and *Akt* exert feedback on the production of two different phosphorylated forms of *IRS*1 (*IRS*1*SP* and *IRS*1*P*). The strength of this feedback is encapsulated by parameters *f*_*PKCZP*_ and *f*_*AktP*_, respectively. See Methods for details. **b)** The temporal dynamics of the input signal (vertical axis), which consists of phosphorylated insulin receptor in response to a 15 minute long insulin pulse. This signal promotes the production of *IRS*1*P* (see equation 2). **c)** Signaling output, represented as a histogram of the distribution of glucose uptake in arbitrary units (a.u.) within a 60 minute interval after the insulin pulse. Blue and red histograms are based on 100 randomly sampled parameter sets that lead to glucose uptake rates characteristics for normal (blue) and diseased, insulin resistant cells (red), based on observed glucose uptake in mouse adipocytes [47]. The inset shows the concentration of membrane-bound *GLUT*4 as a function of time, for the same two randomly sampled parameter sets.

My starting point is a widely used experimentally validated ordinary differential equation model of the core insulin signaling pathway [15]. To render parameter space sampling tractable I simplified this model without sacrificing its dynamical features, considering only events downstream of the insulin-receptor interaction. Briefly, the simplified model captures the following events and processes (Fig. 1a). Insulin-bound insulin-receptor interacts with *IRS*1 (insulin receptor substrate 1), which becomes tyrosine-phosphorylated. Phosphorylated *IRS*1 (denoted as *IRS*1*P* in the model) helps activate phosphoinositide 3-kinase (*PI*3*K*), which phosphorylates phosphatidylinositol (4, 5)-bisphosphate (*PI*45*P*2) to produce the second messenger phosphatidylinositol (3, 4, 5)-triphosphate (*PI*345*P*2). Unless the latter molecule is dephosphorylated by the phosphatase *PTEN* (phosphatidylinositol-3, 4, 5-trisphosphate 3-phosphatase) to *PI*45*P*2, or by the phosphatase *SHIP* (*SH2* domain-containing inositol 5′-phosphatase)) to phosphatidylinositol (3, 4)-bisphosphate (*PI*34*P*2), it binds to a number of effector molecules, among them the protein kinase *Akt* (also known as protein kinase B), and protein kinase C-*ζ* (*PKCZ*). In response, both molecules become phosphorylated (*AktP* and *PKCZP* in the model), and contribute to the translocation of the glucose transporter *GLUT*4 from intracellular compartments (*GLUT*4_*int*_) to the plasma membrane (*GLUT*4_*mem*_), which enables the cell to import glucose. *AktP* also exerts positive feedback on insulin signaling, by inhibiting the ability of *PTP*1*B* (phospho-tyrosine protein phosphatase 1B) to dephosphorylate *IRS*1*P* and the insulin receptor [45]. Moreover, *PKCZP* exerts negative feedback by phosphorylating serine residues on *IRS*1, which can reduce the concentration of *IRS*1*P*, and thus the activation of *PI*3*K* [46].

### Model structure

Each of the state variables of the model’s 14 ordinary differential equations represents the amount or proportions of a molecule through square brackets enclosing molecule abbreviations from the preceding paragraph. Amounts are given in nanomoles (nM) for compounds *IRS*1, *IRS*1*P, IRS*1*SP, PI*3*K*, and for the complex of the latter two, denoted as *IRS*1*P*.*PI*3*K*. All other state variables are given in percentages, such that the following sums are equal to 100 percent: *Akt*+*AktP, PKCZ*+*PKCZP, GLUT*4_*int*_+*GLUT*4_*mem*_. Rate parameters for mass-action reactions are written in lowercase *k*, subscripted with *F* and *B* for forward and backward reaction rates, respectively. Those parameters that are subject to parameter space sampling are written in bold type.

The model’s first three equations are

$$\begin{array}{c} \frac{d[IRS1]}{dt}=-k_{F,IRS1P}[IRS1][SIGNAL]+f_{AktP}k_{B,IRS1P}[Akt][IRS1P]\\ +k_{B,IRS1SP}[IRS1SP]-f_{PKCZP}[PKCZP][IRS1] \end{array}$$ (1)

$$\begin{array}{cc} \frac{d[IRS1P]}{dt} & =k_{F,\mathrm{IRS}1P}\left[IRS1\right]\left[SIGNAL\right]-f_{\mathrm{AktP}}k_{B,\mathrm{IRS}1P}\left[Akt\right]\left[IRS1P\right]\\ & +k_{B,\mathrm{IRS}1P.\mathrm{PI}3K}\left[IRS1P.PI3K\right]-k_{F,\mathrm{IRS}1P.\mathrm{PI}3K}\left[PI3K\right]\left[IRS1P\right] \end{array}$$ (2)

$$\begin{array}{cc} \frac{d[IRS1SP]}{dt} & =f_{\mathrm{PKCZP}}\left[PKCZP\right]\left[IRS1\right]-k_{B,\mathrm{IRS}1\mathrm{SP}}\left[IRS1SP\right] \end{array}$$ (3)

These equations incorporate the action of insulin on changes in the concentration of *IRS*1. The *SIGNAL* term corresponds to the concentration of insulin receptors singly or doubly phosphorylated in response to insulin, which is the upstream input for the model considered here (and is equivalent to the sum of state variables *x*_4_+*x*_5_ in [15]). The left-most terms on the right-hand sides of equations (1) and (2) reflect the increase in tyrosine-phosphorylated *IRS*1, i.e., of *IRS*1*P*, as a result of this signal. The second terms reflect the positive feedback of *AktP* on the production of *IRS*1*P*. This feedback is mediated by *PTP*1*B*, which is not directly modeled here, but through a dimensionless feedback parameter *f*_*Akt*_ which reflects the feedback strength. The terms corresponding to this feedback are simple mass action terms. Note that this feedback is expressed as an increase in *IRS*1, and although *Akt* and not *AktP* appears in the pertinent expressions, *AktP* = 100−*Akt*, implying that *IRS*1*P* will show an increase that is proportional to *AktP*.

The two major terms on the right-hand side of equation (3) reflect the creation of serine-phosphorylated *IRS*1, i.e., *IRS*1*SP*, by *PKCZP*, whose rate is modeled by the dimensionless feedback parameter *f*_*PKCZP*_, and to the conversion of *IRS*1*SP* into *IRS*1. These terms are matched by the third and fourth right-hand terms of equation (1).

Equations 4 and 5 below reflect the association and dissociation of *IRS*1*P*, the active *IRS*1 moiety in this model, with *PI*3*K* in the complex *IRS*1*P*.*PI*3*K*.

$$\begin{array}{cc} \frac{d[PI3K]}{dt} & =k_{B,\mathrm{IRS}1P.\mathrm{PI}3K}\left[IRS1P.PI3K\right]\\ & -k_{F,\mathrm{IRS}1P.\mathrm{PI}3K}\left[PI3K\right]\left[IRS1P\right] \end{array}$$ (4)

$$\begin{array}{cc} \frac{d[PI3K.IRS1P]}{dt} & =k_{F,\mathrm{IRS}1P.\mathrm{PI}3K}\left[PI3K\right]\left[IRS1P\right]\\ & -k_{B,\mathrm{IRS}1P.\mathrm{PI}3K}\left[IRS1P.PI3K\right] \end{array}$$ (5)

The following equations (6), (7), and (8) encapsulate the creation of *PI*345*P*3 from *PI*45*P*2, and its decay into *PI*45*P*2 or *PI*34*P*2. Although the dephosphorylation of *PI*345*P*3 is promoted by the phosphatase *PTEN*, the concentration of this phosphatase is not modeled directly here. Its action is instead encapsulated in the parameter *k*_*B, PI*45*P*2_. Likewise, the action of the phosphatase *SHIP* to promote the decay of *PI*345*P*3 to *PI*34*P*2 is encapsulated in *k*_*B, PI*34*P*2_. Note that these three equations obey mass balance, such that the sum of the relative amounts of the three phospholipids is constant.

$$\begin{array}{ll} \frac{d[PI345P3]}{dt} & =k_{F,\mathrm{PI}45P2}\left[IRS1P.PI3K\right]\left[PI45P2\right]+k_{F,\mathrm{PI}34P2}\left[PI34P2\right]\\ & -\left(k_{B,\mathrm{PI}45P2}+k_{B,\mathrm{PI}34P2}\right)\left[PI345P3\right] \end{array}$$ (6)

$$\begin{array}{cc} \frac{d[PI45P2]}{dt} & =k_{B,\mathrm{PI}45P2}\left[PI345P3\right]-k_{F,\mathrm{PI}45P2}\left[IRS1P.PI3K\right]\left[PI45P2\right] \end{array}$$ (7)

$$\begin{array}{cc} \frac{d[PI34P2]}{dt} & =k_{B,\mathrm{PI}34P2}\left[PI345P3\right]-k_{F,\mathrm{PI}34P2}\left[PI34P2\right] \end{array}$$ (8)

Equations (9)–(12) describe the phosphorylation (stimulated by *PI*345*P*3) and dephosphorylation of the proteins *Akt* (9–10) and *PKCZ* (11–12).

$$\begin{array}{cc} \frac{d[Akt]}{dt} & =k_{B,\mathrm{AktP}}\left[AktP\right]-k_{F,\mathrm{AktP}}\left[PI345P3\right]\left[Akt\right] \end{array}$$ (9)

$$\begin{array}{cc} \frac{d[AktP]}{dt} & =k_{F,\mathrm{AktP}}\left[PI345P3\right]\left[Akt\right]-k_{B,\mathrm{AktP}}\left[AktP\right] \end{array}$$ (10)

$$\begin{array}{cc} \frac{d[PKCZ]}{dt} & =k_{B,\mathrm{PKCZP}}\left[PKCZP\right]-k_{F,\mathrm{PKCZP}}\left[PI345P3\right]\left[PKCZ\right] \end{array}$$ (11)

$$\begin{array}{cc} \frac{d[PKCZP]}{dt} & =k_{F,\mathrm{PKCZP}}\left[PI345P3\right]\left[PKCZ\right]-k_{B,\mathrm{PKCZ}}\left[PKCZP\right] \end{array}$$ (12)

Both *AktP* and *PKCZP* promote the translocation of glucose transporters from the cell’s interior (*GLUT*4_*int*_) to the membrane (*GLUT*4_*mem*_). I here follow [15], which assumes that receptors are translocated to the membrane at a basal (non-insulin-stimulated) rate determined by a parameter *k*_13_ [15], and internalized at a different basal rate (*k*_−13_), such that in the absence of insulin stimulation, only four percent of receptors are membrane-localized. Insulin-stimulated translocation to the cell membrane proceeds at a rate *k*_13^′^_ chosen such that at maximum stimulation 10 times more receptors (40 percent) are membrane-localized than in the unstimulated state. Receptors are synthesized and decay at rates *k*_14_ and *k*_−14_. The resulting equations (using the parameter names from [15]) are

$$\begin{array}{ll} \frac{d[GLUT4_{int}]}{dt} & =k_{-13}\left[GLUT4_{mem}\right]-\left(k_{13}+k_{13\prime }\right)\left[GLUT4_{int}\right]\\ & +k_{14}-k_{-14}\left[GLUT4_{int}\right] \end{array}$$ (13)

$$\begin{array}{cc} \frac{d[GLUT4_{mem}]}{dt} & =\left(k_{13}+k_{13\prime }\right)\left[GLUT4_{int}\right]-k_{-13}\left[GLUT4_{mem}\right] \end{array}$$ (14)

Because *k*_13^′^_ is a parameter that reflects insulin stimulation, it must depend on the concentrations of *AktP* and *PKCZP*. This dependency is given by *k*_13^′^_ = [(40/60)−(4/96)]*k*_−13_(0.2[*PKCZP*] + 0.8[*AktP*])/*AP*_*eq*_, where *AP*_*eq*_ is equal to [*AktP*] + [*PKCZP*] at maximal insulin stimulation. See [15] for further details on this expression. In my analysis, I did not vary the parameters in (13) and (14) but used only their published values, which are *k*_13_ = 0.006958min^−1^, *k*_−13_ = 0.167min^−1^, *k*_14_ = 0.11088min^−1^, *k*_−14_ = 0.001155min^−1^, *AP*_*eq*_ = 9.09% [15].

Together, equations (1)–(12) form the core model I analyze. That is, I subject all their parameters to parameter space sampling. Equations (13) and (14) link this core to the pathway output, namely the concentration of membrane-localized glucose transporter, which determines the glucose uptake rate.

The molecular components of this model are of demonstrable relevance to type 2 diabetes [48]. For example, *IRS*1 tyrosine phosphorylation and its interaction with *PI*3*K* is impaired in skeletal muscle of type 2 diabetes patients [48, 49]. Activation of *PKCZ* by *PI*345*P*3 is impaired in muscle tissues of type 2 diabetes patients [48, 50]. And while *GLUT*4 concentrations are not necessarily altered in type 2 diabetes, *GLUT*4 translocation is impaired [51].

However, it is also important to note that this model represents only a figment of the true complexities of insulin signaling [16, 37, 48, 52–54]. (Every one of these complexities would further increase the potential for causal drift, as it would add additional parameters that can vary without causing the phenotype to vary.) First, the model does not represent some molecular interactions explicitly, such as that between *AktP* and *PTPB* [45], but encapsulates them in a parameter. Second, it ignores the indirect nature of some interactions, such as that between *PI*3*K* and *Akt*, which is mediated by *mTOR* (mammalian target of rapamycin), or that between *AktP* and *GLUT*4 translocation, which is mediated by proteins *AS*160 and *RabGTPase* [48, 55]—it represents the latter through phenomenological terms in (13) and (14). Third, the model does not explicitly represent the multiple phosphorylation sites on *IRS*1 (see [Fig. 4] of [48] and [56]). Fourth, it neglects some pathway components, such as *IRS*2 [57]. Fifth, it does not incorporate cross-talk to other important signaling pathways, such as the Erk (extracellular signal-related kinase) pathway [16, 58]. Sixth, on a higher level of organization, the model does not represent interactions between different organs relevant for glucose homeostasis, such as that between the brain and pancreas [38]. Seventh, the model does not consider aspects of glucose homeostasis different from glucose uptake, e.g., the regulated synthesis of glycogen, which is also mediated by *Akt* and one of its targets, glycogen synthase kinase 3 [59]. Finally, the model does not incorporate possible gene expression changes of signaling proteins. More detailed models may give a more comprehensive view of insulin signaling [14, 16, 52–54]. However, their complexity (e.g., more than 100 parameters in [16]) makes parameter space sampling impossible, because the computational cost of such sampling increases exponentially with the number of parameters [23].

### Input signal, initial states, and parameter values

The upstream signal that serves as the model input is the concentration of insulin-bound insulin receptor in response to a (rectangular) pulse of 100 nM insulin that lasts from *t* = 0 to *t* = 15min. This response, encapsulated in the variable *SIGNAL* is shown in Fig. 1b. It shows a sharp increase of insulin-bound receptor from a value of zero to a value close to 0.9nM in less than a minute after insulin exposure, followed by a 15 minute plateau and a fast decay after insulin removal at 15mins. Although I used this specific insulin input primarily to ensure consistency and comparability with the previous model [15], I note that its time scale of insulin administration and response are consistent with experimental work [14, 47].

The initial values of other state variables at time *t* = 0 reflect the assumption that before insulin stimulation, (i) the concentration of active, phosphorylated *IRS*1 is negligible ([*IRS*1](0) = 1nM, [*IRS*1*P*](0) = 0nM, [*IRS*1*SP*](0) = 0nM) [60, 61], (ii) the concentration of the active PI3K-IRS1P complex is zero ([*PI*3*K*](0) = 0.1nM, [*IRS*1*P*.*PI*3*K*](0) = 0nM) [62, 63], (iii) most of the relevant signaling phospholipids exist in the form of *PI*45*P*2 ([*PI*345*P*3] = 0.31%, [*PI*45*P*2] = 99.4%, [*PI*34*P*2](0) = 0.29%) [64], (iv) all of *Akt* and *PKCZ* exist in their inactive forms ([*Akt*](0) = 100%, [*AktP*](0) = 0%, [*PKCZ*](0) = 100%, [*PKCZP*](0) = 0%), and (iv) the vast majority of glucose transporters *GLUT*_4_ exists in the inactive, intracellular form ([*GLUT*4_*int*_](0) = 96%, [*GLUT*4_*mem*_](0) = 4%) [65, 66]. Because absolute molecular concentrations given by [15] ( ≈ 10^−15^M) are some three orders of magnitude too low given today’s knowledge about eukaryotic cell volumes [67–70], I rescaled these concentrations by a factor 1000, and rescaled parameters depending on concentrations [15] accordingly: *k*_*F, IRS*1*P*_ = 10min^−1^nM^−1^, *k*_*B, IRS*1*P*_ = 5min^−1^, *k*_*B, IRS*1*SP*_ = 0.1min^−1^, *k*_*F, IRS*1*P*.*PI*3*K*_ = 0.706min^−1^nM^−1^, *k*_*B, IRS*1*P*.*PI*3*K*_ = 10min^−1^, *k*_*F, PI*45*P*2_ = 300min^−1^nM^−1^, *k*_*B, PI*45*P*2_ = 42.15min^−1^, *k*_*F, PI*34*P*2_ = 2.96min^−1^, *k*_*B, PI*34*P*2_ = 2.77min^−1^, *k*_*F, AktP*_ = *k*_*F, PKCZP*_ = 0.21min^−1^, *k*_*B, AktP*_ = *k*_*B, PKCZP*_ = 6.93min^−1^, *f*_*AktP*_ = 0.001, *f*_*PKCZP*_ = 0.3. With these parameter values, the simplified signaling model I use can reproduce the state variables’ temporal dynamics from [15], some of which have been experimentally validated.

### Parameter space sampling

Only some of the values of the 15 biochemical parameters I subject to sampling (bold type in (1)–(12)) have been measured [15]. Because the model’s parameter values span approximately six orders of magnitude, i.e., the interval (10^−3^, 10^3^) [15], I allowed each of the 15 parameters to assume values within this interval. I sampled in the logarithmic domain, i.e., I created uniformly distributed random variates *x* ∈ (−3, 3), and set the corresponding parameter to 10^*x*^. I refer to each sampled (15-dimensional) point as a parameter set or a parameter vector. I estimated sensitivity coefficients *S* (equation (16)) by imposing a ten percent change in the value of a focal parameter *p* and computing the resulting effect on the glucose uptake rate *U* (defined below in equation (15)).

### Signaling output

The output of the modeled pathway is the concentration of membrane-bound glucose transporter *GLUT*4_*mem*_, because it is proportional to the glucose a cell can import per unit time. This concentration—and thus the rate of glucose uptake—vary over time as a function of changes in other state variables. As a proxy for the total glucose uptake *U* within a one hour time interval after insulin exposure I compute the integral

$$\begin{array}{c} U=\int_{0}^{60}\left[GLUT4_{mem}\right]\left(t\right)dt \end{array}$$ (15)

which is also the molecular phenotype I consider. For the parameter values given above, *U*_*ref*_ ≈ 1.17×10^3^ arbitrary units (a.u.), which I use as a reference for a normal (healthy, wild-type) glucose uptake phenotype. To define a phenotype associated with disease, I took advantage of the experimental observation that insulin-resistant mice with impaired *GLUT*4 expression show an approximately 70 percent reduction in glucose uptake by adipocytes at a concentration of 100nM insulin, i.e., from ≈ 85±35(s.dev.) to ≈ 25±12 attomoles per cell per minute [47]. I translated these figures into the arbitrary units above, allowing one standard deviation below *U*_*ref*_ as the minimally admissible glucose uptake for the normal state, and one standard deviation above 30% of *U*_*ref*_ as the maximally admissible glucose uptake rate for the diseased state. This yields *U*_*normal*_ ≔ *U*_+_ > 691.26 and *U*_*diseased*_ ≔ *U*_−_ < 502.86. In addition, I required for a normal phenotype that *GLUT*4_*mem*_ shows *bona fide* regulation in response to insulin, i.e., after increasing when stimulated with insulin, it needed to decrease upon insulin-removal. Specifically, after [*GLUT*4_*mem*_] had reached a maximum at some time point *t*_*max*_ ∈ (0,60), I required that it falls below one half of this maximum for some *t* ∈ (*t*_*max*_, 60).

### Creation of polymorphic ‘populations’

To create groups of parameter sets (‘populations’) that are not uniformly sampled but derived from and localized near some point ${\vec{p}}_{init}$ in parameter space, I used the following procedure. Starting from ${\vec{p}}_{init}$ (which was itself taken from a uniform sample of viable parameters), I chose one of the 15 parameters in it at random, and altered this parameter by replacing it with a random variate 10^*x*^, where *x* was a uniformly distributed pseudorandom number in the interval (−3,3). In other words, I randomized the value of this parameter. If the resulting new parameter set yielded normal glucose uptake, I retained it. If not, I chose again a random parameter among the 15 parameters in ${\vec{p}}_{init}$ and randomized it in the same way. I repeated this procedure until I had found a new parameter set ${\vec{p}}^{\prime }$ with normal glucose uptake. I then repeated this randomization procedure starting from ${\vec{p}}^{\prime }$ instead of ${\vec{p}}_{init}$, until I had found another parameter set ${\vec{p}}^{\prime \prime }$ with normal glucose phenotype (in which now at most two parameters are altered relative to ${\vec{p}}_{init}$). I repeated this procedure starting from ${\vec{p}}^{\prime \prime }$ and its ‘descendants’ until I had identified a total of 10 viable parameter sets increasingly distant to ${\vec{p}}_{init}$ in parameter space, but all showing normal glucose uptake. After that, I restarted the procedure from ${\vec{p}}_{init}$, until I had created another 10 parameter sets in the same manner, and so on, until I had created 100 such 10-tuples of parameter sets, i.e., a ‘population’ of 1000 related parameter sets.

With such a population in place, I derived from it another set of 1000 parameter points whose members all had pathologically reduced glucose uptake (*U* < *U*_−_). I did so with the following procedure. First, I chose at random a member (parameter set) of the population with normal glucose uptake, and from this parameter set I chose at random one of the 15 parameters. Second, I mutated (randomized) the chosen parameter. Third, I computed whether the population member with the mutated parameter has a glucose uptake phenotype below the disease threshold (*U* < *U*_−_). If so, I kept the mutated population member. I repeated these steps (choosing population members and parameters with replacement) until I had created a population of 1000 individuals. Each of its members has a pathologically reduced glucose uptake, and each is derived from a single individual of the population with normal glucose uptake through mutation of a single, randomly chosen parameter.

For logistic regression, I encoded the glucose uptake phenotype in a binary manner, assigning a value of one and zero to parameter sets associated with a healthy and diseased glucose uptake phenotype, respectively.

### Simulated population evolution

Evolutionary simulations started from a population of 100 identical individuals (parameter sets) derived from a single uniformly sampled parameter point with normal glucose uptake phenotype. To mutate individuals in this population, that is, to randomize individual parameters, I performed the following procedure for each of the population’s individuals. I chose a random one among the 15 parameters and randomized it, that is, I replaced it by a random variate 10^*x*^, where *x* has a uniform distribution on the interval (−3,3). Subsequently, I computed the glucose uptake phenotype of the mutated individual. To select from this population of mutated individuals a new population of equal size in which every member has a normal glucose uptake phenotype (*U* > *U*_+_), I chose at random (with replacement) individuals from the mutated population, and placed them in the new population if they had a normal glucose uptake phenotype, until I had filled the population with *N* = 100 individuals. I repeated this cycle of mutation and selection 500 times, and computed the sensitivity coefficient *S* of each parameter every generation, thus yielding a time series for *S, S*(*t*), in the evolving population. From this time series, I computed the autocorrelation (serial correlation) function *ρ*(*τ*) = *cov*(*S*(*t*), *S*(*t* − *τ*))/*var*(*S*(*t*)), where *cov* and *var* indicate covariance and variance, respectively.

## Results

### Viable parameters comprise a large fraction of parameter space

The model of core insulin signaling I build on reproduces experimental data, such as insulin receptor dynamics, signaling complex dynamics, and glucose uptake in rat adipocytes [15, 71]. Its input signal (Fig. 1a) is insulin-bound insulin-receptor that is formed in response to a 100 nM insulin pulse of 15 minute duration (Fig. 1b). The receptor interacts with the protein *IRS*1 (insulin receptor substrate-1) which becomes tyrosine-phosphorylated. Phosphorylated *IRS*1 interacts with phosphoinositide 3-kinase (*PI*3*K*) to release phosphatidylinositol (3,4,5)-triphosphate (*PI*345*P*2), which activates the protein kinases C-*ζ* (PKCZ) and *Akt* (Fig. 1a, see Methods). The latter two molecules regulate the translocation of the glucose transporter *GLUT*4 from intracellular compartments (*GLUT*4_*int*_) to the plasma membrane (*GLUT*4_*mem*_), where it facilitates glucose import.

Thirteen of the model’s 15 parameters describe the rates of mass-action reactions between signaling molecules, and the remaining two (*f*_*AktP*_ and *f*_*PKCZP*_) describe the strength of two feedback loops in the circuit (see Methods and Fig. 1a). The primary circuit output is the concentration change of *GLUT*4_*mem*_ whose time integral over 60 minutes I use as a proxy of glucose uptake (Fig. 1c). To distinguish normal circuit output (phenotype) from the pathological phenotype associated with insulin-resistance, I take advantage of the observation that insulin-resistant mice with impaired glucose-import show a ≈ 70% reduction in glucose uptake into adipocytes after stimulation with 100 nM insulin [47] (see Methods).

I allowed each of the 15 parameters to range over six orders of magnitude (10^−3^ < *p* < 10^3^) and explored the 15-dimensional parameter space through ‘brute-force’ uniform sampling in the logarithmic domain (see Methods). From a sample of 9.31×10^5^ parameter sets, I computed a viable volume for the normal glucose uptake phenotype of *V*_*n*_ = 0.076±2.7×10^−4^, expressed as a fractional volume of parameter space. (The error term reflects the standard deviation of the volume estimate, based on a normal approximation.) This fractional volume is remarkably high, but not unusually so among other robust circuits [20, 29]. If along each axis of parameter space the same fraction *p* of randomly chosen parameters gave rise to a viable parameter point, the viable volume *V*_*n*_ would be given by *V*_*n*_ = *p* ^15^, and thus *p* = ln *V*_*n*_/15 ≈ 0.84. In other words, some 84 percent of randomly chosen values for each single parameter could yield a normal glucose uptake phenotype (depending on the values of other parameters).

The same sampling procedure yields a fractional volume *V*_*d*_ = 0.33 ± 4.9 × 10^−4^ for the pathological phenotype of reduced glucose uptake (9.31 × 10^5^ sampled points). Because *V*_*d*_/*V*_*n*_ ≈ 4.4, it is four times more likely that a randomly chosen parameter set yields a disease phenotype than a normal phenotype. Not surprisingly then *p* = ln *V*_*d*_/15 ≈ 0.93 is also greater than for normal glucose uptake, meaning that 93 percent of randomly chosen values for any one parameter yield a disease state.

### Parameters associated with normal or impaired glucose uptake have broad and overlapping ranges

**Fig 2:**
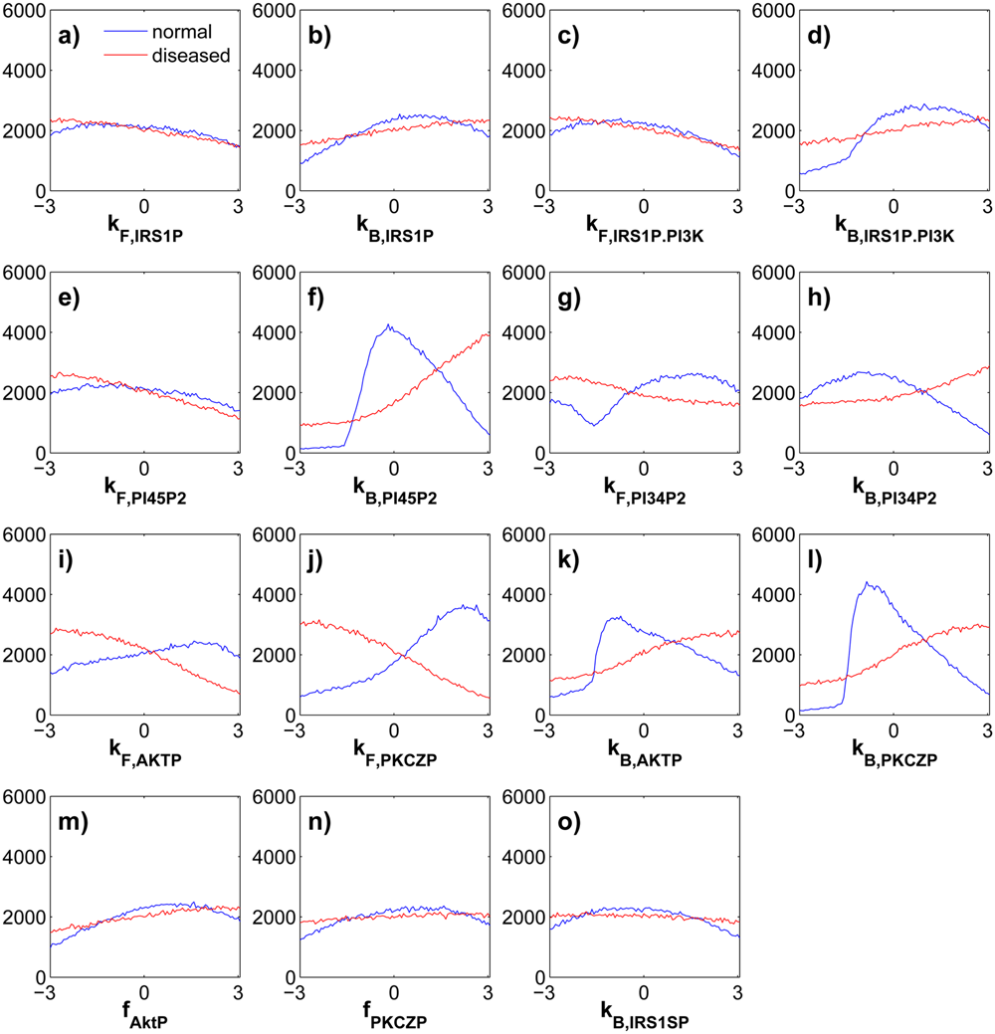
Distribution of randomly sampled parameters that yield normal and diseased signaling behavior. Each panel shows, on a logarithmic horizontal scale, the distribution of parameter values *p* ∈ (10^−3^, 10^3^) that yield normal (blue) or reduced (red) glucose uptake. Each data set in each panel is based on 2×10^5^ parameter sets sampled uniformly from the viable region of parameter space for the two phenotypes.

Computing the fraction *p* of randomly chosen parameters that yield a specific phenotype tacitly assumes that different parameters can assume broad ranges of values. This assumption may be violated if parameters critical for signaling behavior are confined to a narrow interval of parameter space. This is not the case, as Fig. 2 and S1 Fig. show. Most parameters can assume very broad ranges of values, both for normal (blue) and reduced (red) glucose uptake. For example, the distributions of 6 parameters (blue in Fig. 2a-c and Fig. 2m-o) are flat and almost uniform over six orders of magnitude, meaning that all values for the respective parameters are similarly likely to display a specific signaling behavior. Even parameters with evident preferences for some values are not strongly constrained. The most narrowly distributed parameters are *k*_*B, PI*45*P*2_ (Fig. 2f, blue), which promotes the deactivation of the signaling molecule *PIP*345*P*3; and *k*_*B, PKCZP*_ (Fig. 2l), which accelerates dephosphorylation of protein kinase C-*ζ* (Fig. 1a). Their probability distributions are somewhat concentrated over four instead of six orders of magnitude, but even they are not equal to zero outside this parameter range. For example, in some parameter sets that yield normal signaling behavior, these parameters assume their lowest possible value of 10^−3^. S1 Fig. further underscores the breadth of these distributions.

As remarkable as these broad distributions are the modest differences between them for normal (blue) and reduced (red) insulin signaling. If parameters exist where particular values are crucial to determine glucose uptake, one might expect them to show non-overlapping distributions. However, the distributions of all parameters overlap widely (Fig. 2 and S1 Fig.), and the distributions of six parameters are almost congruent (blue and red in Fig. 2a-c and Fig. 2m-o). The most distinct distribution pairs indicate weak preferences for some parameter ranges over others. For example, the parameter *k*_*F, PKCZP*_ which promotes phosphorylation of protein kinase C-*ζ* tends to have higher values in circuits with normal signaling behavior (Fig. 2j). This is not surprising, given that phosphorylated protein kinase C-*ζ* promotes membrane translocation of the glucose transporter. Less easily explained is the observation that the parameter for the reverse reaction, *k*_*B, PKCZ*_ does not show preferentially low but intermediate values in normal signaling circuits (Fig. 2l, blue), and only a weak preference for high values in circuits with reduced signaling (Fig. 2l, red). Similarly, the parameter *k*_*B, PI*45*P*2_ for the reaction inactivating the signaling molecule *PI*345*P*2 does not show low but intermediate values in normal insulin signaling (Fig. 2f, blue), and only a weak preference for high values—which promote reduced glucose uptake—in circuits with the pathological phenotype (Fig. 2f, red).

In sum, the values of individual parameters associated with normal or reduced glucose signaling behavior show very broad and broadly overlapping distributions, indicating no obvious ‘choke-points’ that are generic circuit properties independent of any one parameter set. In the supplementary online material I show that pairwise statistical associations among viable parameters are weak (S2 Fig.). Moreover, a principal component analysis shows that no linear combination of parameters can explain most parameter viability, either for normal or for impaired signaling (S3 Fig.).

### The importance of any one parameter varies by orders of magnitude among viable parameter sets

The parameter(s) in which change is most likely to alter the signaling phenotype are good candidates for genetic determinants of disease. To identify these parameters I used two complementary approaches. In the first I estimated sensitivity coefficients *S*, which indicate by how much the glucose uptake rate phenotype *U* changes (Δ*U*) when a parameter *p* is changed by a small amount (Δ*p*). Specifically,

$$\begin{array}{c} S=\frac{\Delta U/U}{\Delta p/p} \end{array}$$ (16)

Note that *S* is dimensionless, because the numerator and the denominator of this quantity express the amount of change as a ratio, i.e., relative to the current values of *U* and *p*. A value of *S* = 1 indicates that a small change in a parameter’s value will cause an equal amount of change in glucose uptake relative to its current value. For brevity, I will also refer to *S* as the *importance* of the parameter *p* for the phenotype.

**Fig 3:**
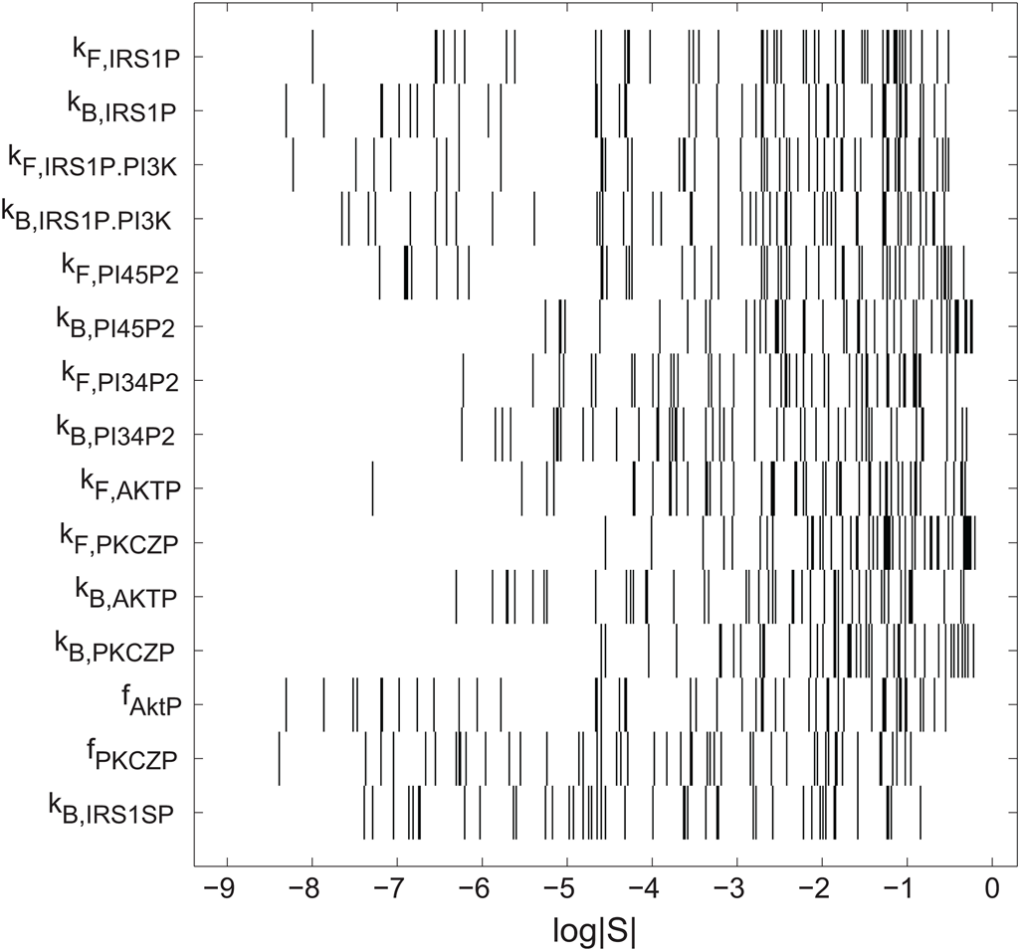
The importance of the same parameter can vary widely across different parameters sets. The horizontal axis indicates the log-transformed absolute value of sensitivity coefficients *S*. Each horizontal row contains 50 black vertical bars, which correspond to the sensitivity coefficient *S* of one parameter (labeled on the vertical axis) for 50 different parameter sets uniformly and randomly sampled from the region of parameter space yielding a normal glucose uptake phenotype. Note that each parameter’s sensitivity coefficient *S* varies over multiple orders of magnitude.

I computed sensitivity coefficients for all 15 parameters in each of 1000 uniformly sampled parameter sets that yield normal circuit behavior. The same parameter can vary dramatically in its importance, depending on the viable parameter set that it is a part of. Fig. 3 indicates the distribution of sensitivity coefficients (horizontal axis) for each of the 15 model parameters, based on 50 different uniformly sampled parameter sets. Each vertical bar indicates the sensitivity of the phenotype to a small parameter change. Note the logarithmic horizontal axis, which shows that any one parameter can vary in its impact on the phenotype by several orders of magnitude. (I note parenthetically that the signaling circuit also shows sloppy control [22], i.e., *different* parameters in the same parameter set also vary in their importance (S4 Fig.).)

The full sample of 1000 parameter sets demonstrates the very broad range of *S*, which spans more than five orders of magnitude for all parameters, and more than ten for several parameters, such as *k*_*F, IRS*1*P*.*PI*3*K*_ (S5 Fig. and S6 Fig.). Any given parameter may have close to the maximal influence on glucose uptake for one parameter set (∣*S*∣ ≈ 0.5), and a negligible influence (*S* ≈ 10^−5^−10^−10^) in some other parameter set (S6 Fig.).

**Fig 4:**
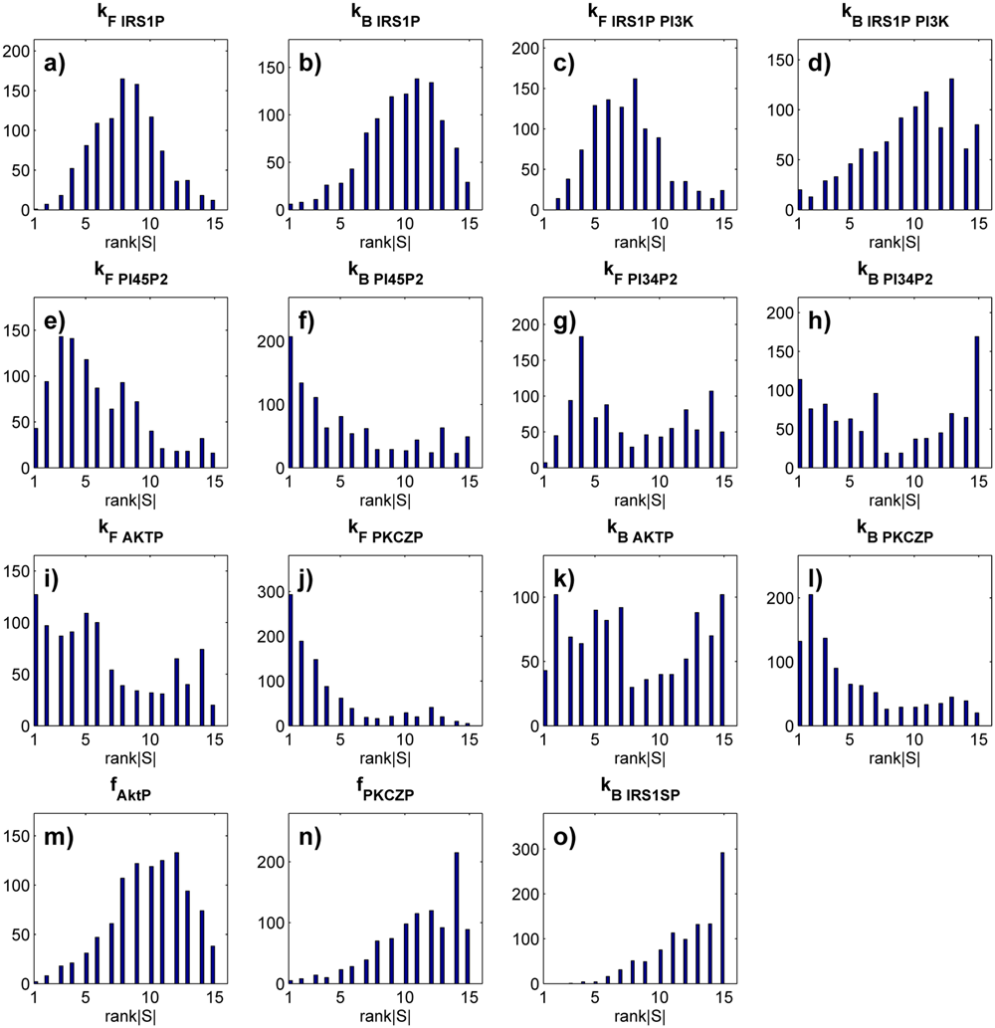
A parameter’s importance depends strongly on the parameter set. The data is based on sensitivity coefficients of all parameters in each of 1000 uniformly sampled parameter sets that yield a normal glucose uptake phenotype. For each of the 1000 viable parameter sets, I ranked parameters according to the magnitude of the absolute value of their sensitivity coefficient, from rank one (largest ∣*S*∣) to rank 15 (smallest ∣*S*∣). Each panel shows, for the parameter indicated on top, a histogram of the distribution of these ranks among the 1000 parameter sets. The vertical axis is drawn on a linear scale. Note the broad distribution of parameter ranks, implying that a parameter’s importance can vary broadly among parameter sets.

Next I asked how the importance of the parameters changes relative to one another among different parameter sets. To this end, I ranked the parameters according to the magnitude of the absolute value of *S* for each parameter set. The most important parameter (that with the largest sensitivity coefficient *S*) received rank one and that with the smallest *S* received rank 15. I then analyzed the distribution of these ranks across all 1000 uniformly sampled parameter set. This distribution is very broad (Fig. 4). Specifically, for all but two of the 15 parameters, the distribution spans the entire range from 1 to 15. That is, any one of these parameters is the most important (it has the greatest effect on glucose uptake) for some parameter set, the least important in some other parameter set, and it has intermediate importance in others. The ranks of the remaining two parameters *k*_*F, IRS*1*P*.*PI*3*K*_ and *k*_*B, IRS*1*SP*_ (Fig. 4c and Fig. 4o) range from 2 to 15 and from 3 to 15, respectively. That is, they are at best the second- and third-most important parameters in the circuit. One might think that parameters with a nearly uniform distribution in the viable set (e.g., *k*_*F, PI*45*P*2_ in Fig. 2e) may be of less overall importance than parameters with a more sharply peaked distribution, because they are about equally likely to assume any one value. However, even such parameters can have rank one (Fig. 4e).

### Every parameter also varies broadly in its propensity to cause deleterious phenotypic change when mutated

Sensitivity coefficients are calculated from small parameter changes, but DNA mutations may cause much large changes in the parameters of a biochemical system. I next asked whether the effects of larger parameter changes are as variable as sensitivity coefficients. To this end, I repeated the following procedure 100 times for each of the 15 parameters *p* in each of 1000 uniformly sampled parameter sets that yield normal glucose signaling: I randomized (‘mutated’) *p* by assigning to it a randomly chosen new value that was uniformly distributed along the sampling interval (10^−3^, 10^3^), and computed the resulting glucose uptake phenotype. If the change led to glucose uptake below the disease threshold, I called the change deleterious. (S7 Fig. illustrates that the majority of mutations cause small changes in glucose uptake.) From this data, I computed the fraction *f*_*del*_ of deleterious mutations in each parameter. For each parameter and across the 1000 parameter sets, *f*_*del*_ shows a highly significant correlation to *S*, but one that is only modest in value for some parameters (Spearman’s R = 0.16–0.76, *P* < 1.7×10^−7^, *n* = 1000 for all parameters), illustrating that *S* cannot generally substitute as a measure of *f*_*del*_. The distribution of *f*_*del*_ shows two commonalities across all parameters. First, in the vast majority of parameter sets, none of the 100 mutations in any one parameter have a deleterious effect on glucose uptake, a reflection of the robustness of this signaling circuit (S8 Fig.). Second, for any one parameter *f*_*del*_ strongly depends on the parameter set and ranges from zero to more than one half (more than 50 percent of mutations are deleterious, S8 Fig.). Similarly, a parameter’s rank in its propensity *f*_*del*_ to suffer deleterious mutations varies broadly across parameter sets (S1 Text, S9 Fig.).

In sum, the impact on phenotype of small (*S*) and large (*f*_*del*_) parameter changes shows similar patterns. No one determinant of glucose uptake phenotype is consistently more important than others. Its importance crucially depends on the genetic background one considers.

### Logistic and linear regression analysis also demonstrate shifting importance of parameters

Identification of genetic disease determinants often relies on case-control studies, in which many individuals that are healthy (controls) or affected by a disease (cases) are genotyped genome-wide, and genes or genetic markers associated with the disease are identified with statistical methods. A frequently used such method is logistic regression, a generalization of linear regression suitable to analyze data from case-control studies, because it uses binary dependent variables (afflicted/normal phenotype). The logistic regression coefficient *β*_*i*_ of any one candidate predictor variable *l*_*i*_ (such as a nucleotide polymorphism) on disease state can be interpreted as follows: A one unit increase in the predictor variable *l*_*i*_ alters the logarithm of the odds-ratio of getting a disease by *β*_*i*_ [72]. Predictor variables with larger *β*_*i*_ thus alter the odds-ratio to a greater extent and are thus more important in this sense. Statistical tests that ask whether *β*_*i*_ is significantly different from zero can be used as a measure of importance through the *p*-value they generate, because predictors with larger ∣*β*_*i*_∣ will usually have a smaller (more significant) *p*-value. A parameter with smaller *p*-value is more important in this sense.

I performed logistic regression on the glucose uptake phenotype, using a binary classification of this phenotype to distinguish ‘cases’ and ‘controls’ (see Methods). The predictor variables in this analysis were the insulin signaling circuit’s parameters, which are the closest proxies for genetic determinants in a mechanistic model. Because case-control studies are usually performed on individuals that are related by common ancestry in complex ways, the uniformly sampled parameter sets I used in previous analyses would not be appropriate for this analysis. Instead, I started from a single, ‘ancestral’ parameter set and created from it a ‘population’ of related parameter sets (individuals) whose glucose uptake was either normal (1000 individuals) or reduced below the disease threshold (another 1000 individuals, see Methods). I repeated this procedure 100 times, thus creating 100 pairs of case-control populations, and asked how strongly the importance of individual parameters, as represented through the *p*-value of their logistic regression coefficient, varied among them.

**Fig 5:**
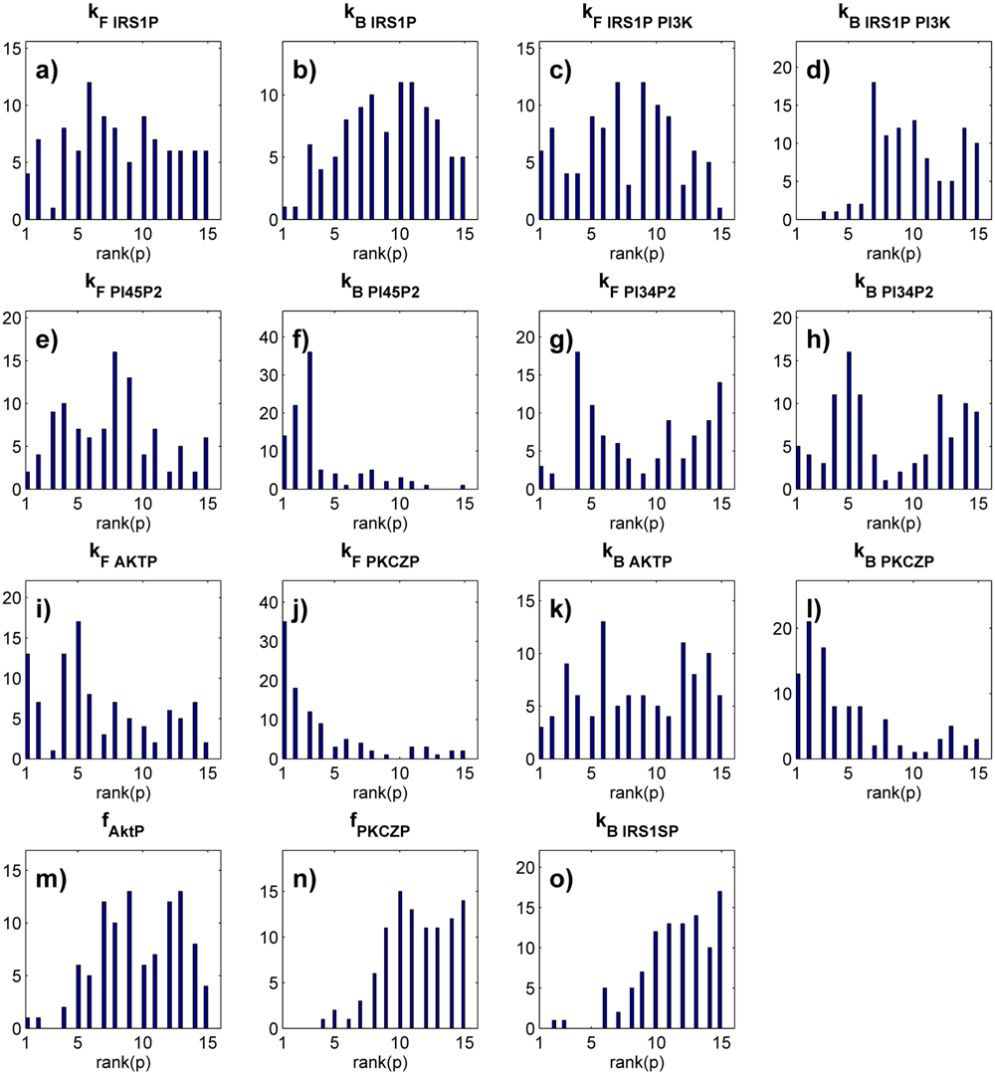
Logistic regression shows that a parameter’s importance for the disease phenotype can vary broadly among populations. Each panel shows a histogram of the rank of *p* for the parameter indicated on top, where *p* is the *p*-value of the parameter’s regression coefficient in a logistic regression against individuals with normal (‘control’) and impaired (‘case’) glucose uptake phenotype. For each parameter set, the parameters were ranked according to the magnitude of *p*, such that the parameter with the smallest *p* (most significant parameter) received the highest possible rank of one, and that with the largest *p* received the lowest possible rank of 15. The vertical axis is drawn on a linear scale. Note that the ranks have a broad distribution for all parameters, indicating that parameters that are important in some individuals are unimportant in others. Data are based on 100 pairs of populations (parameter sets) that showed either normal or reduced glucose uptake. Each population was derived from a single individual with normal glucose uptake and comprised 1000 individuals each (See methods for details). Control and case phenotypes are binarily encoded as one and zero, respectively.

The importance of individual parameters varies enormously (S10 Fig.). Even in the parameter with the least variable importance, as indicated by −*log*_10_ *p* (*f*_*PKCZP*_; S10 Fig., panel n), −*log*_10_ *p* varies over 47 orders of magnitude between *p* = 1.8×10^−48^ to *p* = 0.93. In the most variable parameter (*k*_*F, PKCZP*_, the value of −*log*_10_ *p* varies over 98 orders of magnitude. And while every parameter is very important (has low *p*-value) in some parameter sets, it is completely unimportant in others. The percentage of populations where *p* > 0.05 ranges from 48 percent for *k*_*B, IRS*1*SP*_ (S10 Fig., panel o) to 1 percent for *k*_*B, PI*45*P*2_ (S10 Fig., panel f), with a mean of 24.5 percent among parameters. In other words in an average of one quarter of all populations, any one parameter has no statistically detectable importance to the phenotype, even though it may be the most important determinant of phenotype in other populations. The shifting importance of parameters is also illustrated by the distribution of ranks among the *p*-values, which I computed analogously to similar analyses above. Specifically, for each parameter set in each of the 100 population pairs, I assigned the parameter with the smallest (most significant) *p*-value the highest rank of 1, and that with the largest *p*-value the lowest rank of 15 (Fig. 5). The distribution of ranked importance is broad and spans all 15 possible ranks for 12 of the 15 parameters. Any of these 12 parameters is the most important in some populations but the least important in others. The ranks of the remaining three parameters range from 15 to 3 (*k*_*B, IRS*1*P*.*PI*3*K*_, Fig. 5d), 4 (*f*_*PKCZP*_, Fig. 5n), and 2 (*k*_*B, IRS*1*SP*_, Fig. 5o). That is, these parameters are not the most important in any population.

*Logistic* regression analysis can identify genetic disease determinants when only qualitative phenotypic information (normal/diseased) is available. However, whenever quantitative information is available, such as in the form of glucose uptake rate values, *linear* regression analysis is preferable, because it uses all available phenotypic information. I thus repeated the preceding analysis of *p*-values derived from logistic regression, but for *p*-values derived from linear regression analysis of the 15 parameters against the glucose uptake phenotype, with very similar results (S1 Text, S11 Fig. and S12 Fig.). Briefly, the importance of most parameters for the phenotype varies broadly, from least to most important. Linear regression analysis also demonstrates that in most populations, the majority of phenotypic variance is accounted for by additive interactions among parameters (*R* ^2^ > 0.5, panel a of S13 Fig.), and that the role of pairwise epistasis is minor (S1 Text, panels b and c of S13 Fig.). However, both observations illustrate how linear regression can mislead, because of the broad variability of parameter importance. The relative importance of predictor variables (parameters) in a truly linear model would be essentially the same in different populations of the size I study, but this is emphatically not the case in the insulin signaling circuit, where a parameter may have a very high importance in some populations and very low importance in others.

### Parameter importance fluctuates rapidly in time

**Fig 6:**
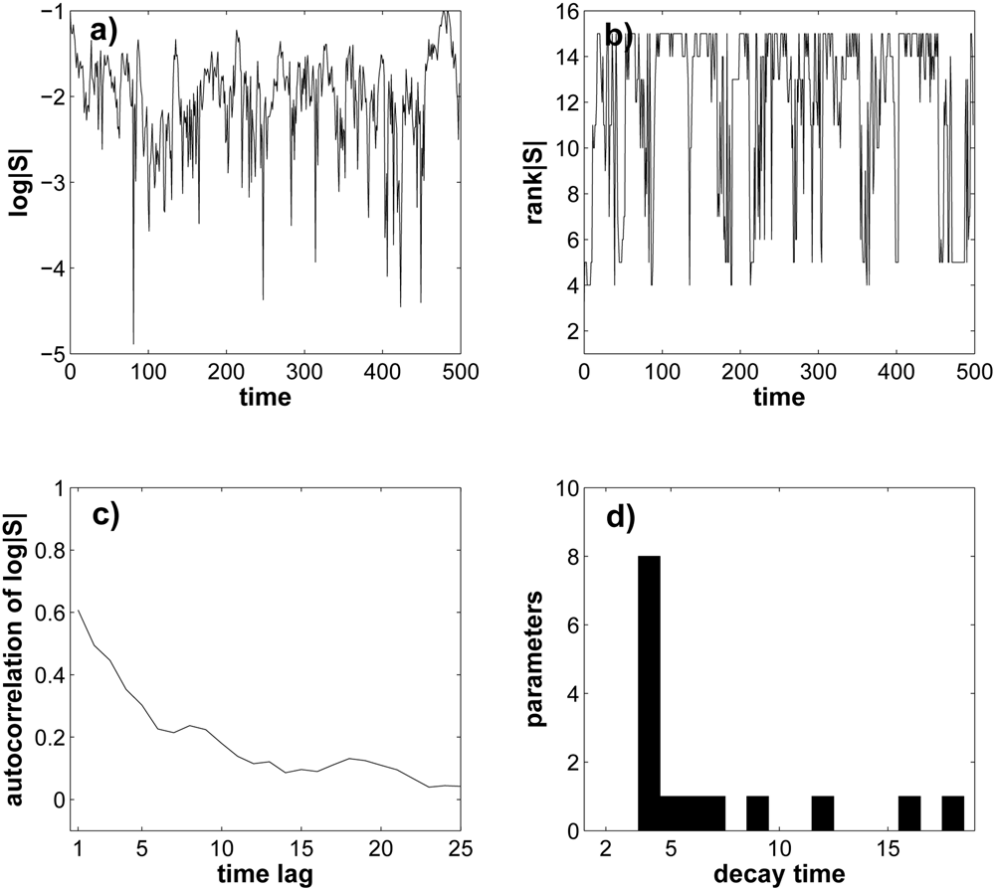
In evolving populations parameters can fluctuate rapidly in their importance. **a)** Temporal change of the sensitivity coefficient (∣*S*∣) for the parameter *k*_*B, PI*34*P*2_ during 500 generations of simulated evolution in a population of *N* = 100 individuals (see Methods). Note the logarithmic vertical scale and the rapid fluctuations. **b)** like a), but for the rank that ∣*S*∣ of *k*_*B, PI*34*P*2_ has among all 15 parameters. **c)** shows the autocorrelation function of *log*∣*S*∣ (vertical axis) as a function of the lag *τ* (see Methods), calculated over 500 generations of simulated evolution. **d)** histogram of decay time for the autocorrelation function of all 15 parameters. The shortest possible decay time given the sampling interval of one generation for the computation of ∣*S*∣ is *τ* = 2.

The observations I made so far do not reveal how fast parameter importance would change in an evolving population that is subject to mutations randomizing parameters and stabilizing selection maintaining normal glucose uptake. To find out, I subjected populations of *N* = 100 individuals (parameter sets) to 500 rounds or ‘generations’ of ‘mutation’(one randomized parameter per generation and individual) followed by stringent selection for normal glucose uptake (see Methods). During this simulation, I computed the sensitivity coefficient *S* for all parameters in every generation. Fig. 6a shows as an example the resulting data for parameter *k*_*B, PI*34*P*2_. The parameter’s importance (∣*S*∣) does not stay constant for long, but fluctuates rapidly and broadly, i.e., by more than thousand-fold (Note the logarithmic vertical scale). Fig. 6b shows that the rank of ∣*S*∣ for this parameter also changes repeatedly and rapidly from a minimum of 15 (least important) to a maximum of four (fourth-most important). Thus, while the parameter does not explore its full range of importance (1–15, Fig. 4h) during this short time, its importance varies broadly.

A commonly used measure for the rate of fluctuation in time-series like this is the autocorrelation function *ρ*(*τ*) of a quantity of interest (here, the sensitivity coefficient *S*(*t*)) at time *t* and time *t* − *τ* (see Methods). This function indicates to what extent *S*(*t*) assumes similar values *τ* generations apart. Fig. 6c shows *ρ*(*τ*) of *S*(*t*) for parameter *k*_*B, PI*34*P*2_ as a function of the time lag *τ*. Even for the smallest time lag *τ* = 1 considered, *ρ*(*τ*) ≈ 0.6, far below the theoretically possible maximum of one. In other words, this parameter changes its importance even at the smallest time lag considered here. Moreover, the decay time of the autocorrelation function, that is, the time needed until *ρ*(*τ*) first decreases below one half of its maximal value at *ρ*(1) is only *τ* = 4 generations. Fig. 6d shows a histogram of this decay time for all 15 parameters. It ranges between 4 and 18 generations, with a mean of 7 generations. In sum, in simulated evolving populations of individuals, the importance of individual parameters can change rapidly on an evolutionary time scale.

## Discussion

I computed four complementary measures of how changes in genetically encoded biochemical parameters would impact the glucose uptake phenotype of the insulin signaling pathway. The first of them is the sensitivity coefficient—the amount of phenotypic change caused by a small parameter change. The second is the fraction of random mutations in a parameter that causes deleterious phenotypic change. The third is based on a multiple logistic regression of all parameters against the phenotype. It is the *p*-value of a test of the null-hypothesis that a parameter’s regression coefficient on the phenotype is equal to zero. The fourth is the same as the third, but for multiple linear regression.

All four measures yield the same observation when applied to different and uniformly sampled viable parameter sets that yield normal glucose uptake: Genetic determinants of the phenotype vary broadly in their impact on this phenotype. A parameter that is crucial for the phenotype in one genotypic background—it has the highest sensitivity, the largest fraction of deleterious mutations, or the most significant (lowest) *p*-value in regression—will be unimportant in other backgrounds. When ranking parameters according to their importance from one (most important) to fifteen (least important), most parameters assume all ranks in some genetic backgrounds. Those that do not are usually not the most important in any background.

Parameter sensitivity and mutational effects gauge how a system’s phenotype reacts to perturbation, which is the most reliable way to identify the causes of a system’s phenotype. Together, they show that in a system like that studied here, any polymorphism that is truly causal in one population or genetic background may be acausal in another. Robust systems can diverge genetically without diverging phenotypically, and such divergence can lead to causal drift—shifting patterns of causality among system components. For a robust system like this, no universally useful choke points exist that could serve to control circuit behavior. I note that there are many biological systems [19–22, 29–31] whose robustness is similar to that of the insulin signaling circuit.

One may question the usefulness of linear regression to analyze an obviously highly nonlinear relationship between circuit parameters and phenotype. My primary motivation to use regression is that it is very common in genome-wide association studies [73]. Its results thus help compare the statistical and mechanistic approach, and they can illustrate how the statistical approach can mislead. In most of the 100 populations I studied, the proportion of phenotypic variation explained by linear regression exceeds 50 percent. The facile conclusion would be that additive interactions among genetic determinants predominate in the insulin circuit. However, a comparison of different populations reveals how variable a parameter’s impact on phenotype can be: Its *p*-value can be non-significant (*p* > 0.05) in one population, yet the most significant among all parameters (e.g., *p* < 10^−100^) in another. Because of the large sample sizes considered here (1000 individuals per population) the estimated regression coefficients and associated *p*-values under true additivity would be virtually identical across populations. In reality they differ wildly, meaning that regression analysis only creates the appearance of additivity. True additivity cannot give rise to the causal drift I observe.

Causal drift is clearly a result of non-additive (epistatic) interactions among parameters. In this regard, it is noteworthy that pairwise epistasis between parameters may not suffice to explain the extent of causal drift, because such epistasis explains little phenotypic variation (S13 Fig.). This observation hints at an important role for higher order epistasis, i.e., for interactions among three or more genetic determinants. Moreover, it also hints at a possible limitation of quantitative genetic studies that fail to detect pairwise epistasis for a given phenotype and population. Explaining phenotypic variation might require higher order epistasis, which is difficult to detect with statistical methods, because of the large number of possible interactions among three or more variates. In addition, it is possible that causal drift shifts patterns of epistasis among populations, just as it shifts the causal influence of individual parameters. In this case, parameters that interact epistatically in one population may lack such interactions in another, such that even combinations of genetic determinants may not suffice to explain a phenotype reliably and across populations.

Mechanistic models like the one I analyze have one obvious limitation. They represent genetic disease determinants through biochemical parameters, and not directly on the level of DNA. However, this limitation is also matched by advantages. First, a mechanistic model can provide insights into disease causes that go beyond statistical associations. Second, factors that can complicate the interpretation of genome-wide association studies, such as recessiveness, linkage disequilibrium, environmental change, and population stratification, play no role here. Third, sample sizes are arbitrarily large, such that lack of statistical power is no limitation. Fourth, because the model is deterministic, the influence of every single variable on the phenotype can be made transparent. The mechanistic approach can show that causal drift is a consequence of the system’s genetic architecture, and not an artefact of limited information about the relevant variables.

Because the model I study here is simple, it is important to note opportunities for causal drift would be even greater in more complex models. For example, the model I consider does not include synthesis and decay rates of individual circuit proteins. In a model incorporating either, the phenotypic effect of a mutation that reduces the rate *k* at which two proteins *A* and *B* bind could be neutralized by an increase in the synthesis rate or a decrease in the degradation rate of either. The reason is that under mass-action, the rate at which *A* and *B* bind is a function of the concentration of both molecules and the rate constant *k*, e.g., *k*[*A*][*B*] in its simplest form. Similarly, the model I consider does not consider signaling components that may be encoded by duplicate genes, such as the insulin receptor substrate *IRS*1 with its duplicate *IRS*2 [74, 75]. Here again, changes in one duplicate that increase its concentration or activity could be compensated by opposing changes in the other. More generally, every additional state variable and interaction provides more opportunities for causal drift.

A central observation of this analysis—that a biochemical circuit’s parameters can vary in their importance—could be influenced by additional constraints on a circuit or its parts. For example, the same biochemical circuit may need to operate under different conditions in different tissues, or some molecules in a circuit may be involved in cross-talk to other circuits, constraining the biochemical parameters that permit such cross-talk. Previous work suggests that such additional constraints would have to be very extreme to affect my observations. For example, in each of 17 different biological circuits, constraining phenotypes by requiring that a circuit’s phenotype fit 100 times more (simulated) concentration measurements than parameters yielded similar variation in the importance of biochemical parameters than that observed here [22]. The varying importance of parameters is a general property of circuit architecture rather than of a specific set of parameters or constraints [22, 35, 76]. In addition, experimental evidence shows that even biological circuits with highly constrained phenotypes can change their architecture. For example, in the circuits controlling galactose metabolism, mating, and ribosomal gene expression of yeast [77–79], the same phenotype can be produced by circuits in which both biochemical parameters and circuit topology vary widely. Another particularly well-studied example comes from the reproductive organs of the nematode worm *C. elegans*, and specifically from the development of its vulva. Different worm species produce morphologically identical adult vulvae but do so through developmental pathways that differ on every level of organization, from the identity and interactions of signaling molecules, to the communication processes between cells in the developing organ [80–85]. It is the flexibility of biological circuits in achieving the same ends by different means that permits the genetic changes responsible for causal drift.

Although we cannot currently estimate the rate at which parameters of biochemical circuits change in human evolution, it is clear that the human population contains ample genetic polymorphisms that affect such parameters. A case in point are genetic polymorphisms in the insulin signaling pathway with demonstrable effects on diabetes and other diseases. For example, a glycine to arginine change in *IRS*1 (G972R) causes a reduction in the ability of *IRS*1 to interact with *PI*3*K*, as well as in the activity of *PI*3*K* [39]. The polymorphism occurs naturally in the human population and is associated with variation among individuals in insulin secretion, and insulin resistance [40–43, 86]. It also affects birth weight in a Brazilian population [87], in line with known effects of insulin signaling on growth phenotypes [88, 89]. In addition, it affects the incidence of polycystic ovary syndrome among Japanese individuals [90] and the risk of colorectal cancer in a Czech population [91]. Similarly, an amino acid change (M326I) in the p85 subunit of *PI*3*K* affects glucose tolerance [41]. Naturally occurring polymorphisms in *PI*3*K, Akt*, and *IRS*2 affect risk for coronary artery disease, metastatic lung cancer, and survival in esophagal cancer [92–94]. Moreover, some such effects depend on the population studied. A G1057D polymorphism in *IRS*2 is associated with diabetes risk in Han Chinese [44], but not with insulin resistance or secretion in a Finnish population [95].

Other experimental evidence also suggests that causal drift exists in the human population. For example, it has long been known that the detrimental effect of a given mutation in a ‘disease-causing’ gene depends strongly on an individual’s genetic background. This holds even for ‘monogenic’ diseases like thalassaemias and phenylketonuria [96, 97]. Genome sequencing has multiplied information about such background effects on disease and drug efficacy, which can even occur within a single family [98]. The increasing importance of personalized medicine and pharmacogenomics [98, 99] underscore the prevalence of such background effects. And if causal drift exists in the human population with its recent common ancestry, it is likely to play an even greater role on the time scales that separate humans from model organisms like mice. If so, caution will be necessary when transferring information about biological circuits from model organisms to understand human disease.

## Supporting Information

S1 Fig Box plot of randomly sampled parameters that yield normal and impaired signaling behavior. The two box plots next to each parameter name reflect the parameter values that yield normal (‘N’, blue) and reduced (‘D’ for diseased, red) glucose uptake. The horizontal axis (logarithmic scale) covers the admissible parameter range (10^−3^, 10^3^). The box lot for each parameter is based on 2×10^5^ parameter sets sampled uniformly from a viable region of parameter space (both for normal and reduced glucose uptake). Circles indicate medians, boxes indicate the 25th percentile, *q*_25_, and the 75th percentile, *q*_75_. Whiskers span the interval (*q*_25_ − 1.5(*q*_75_ − *q*_25_), *q*_75_ + 1.5(*q*_75_ − *q*_25_)) corresponding to approximately 99.3 percent coverage of normally distributed data.
(TIF)

S2 Fig Pairwise associations among model parameters. Each data point shows Spearman’s rank correlation coefficient for a pair of parameters, both for normal glucose signaling (horizontal axis) and reduced glucose signaling (vertical axis). Note the axes scales, which extend only to the half-maximal possible values for *R*. Data are based on 1000 pairs of parameter sets sampled uniformly from the region of parameter space associated with normal or reduced glucose signaling.
(TIF)

S3 Fig Principal component analysis. Data are based on 1000 uniformly sampled parameter sets that yield normal (panels a) and b)) or reduced (c) and d)) signaling behavior. Panels **a)** and **c)** show the fraction of the variance explained by each of the 15 principal components (horizontal axis) of the 15-dimensional parameter space. Panels **b)** and **d)** show the first two principal components plotted against one another.
(TIF)

S4 Fig Sensitivity coefficients vary widely among parameters across multiple parameter set. The horizontal axis indicates the log-transformed absolute value of sensitivity coefficients *S*. The plot has 50 horizontal rows of short vertical bars. Each row corresponds to a different parameter set uniformly and randomly sampled from the viable region of parameter space yielding a normal glucose uptake phenotype. Each row has 15 vertical bars, which indicate the value of *S* for each of the 15 parameters in the parameter set (For rows where this number of bars appears lower, two or more parameters have values of *S* that are so similar that some individual bars cannot be resolved). Note that the different parameters have sensitivity coefficients *S* that vary over multiple orders of magnitude.
(TIF)

S5 Fig Sensitivity of phenotype to perturbation of a given parameter varies broadly among viable parameter sets. Each panel shows the distribution of sensitivity coefficients for the parameter indicated on top, based on 1000 randomly and uniformly distributed parameter sets that yield a glucose uptake phenotype. Note the logarithmic vertical axis.
(TIF)

S6 Fig Sensitivity of phenotype to parameter perturbation varies broadly among viable parameter sets. Each panel shows the distribution of *log*∣*S*∣, the decadic logarithm of the absolute value of sensitivity coefficients for the parameter indicated on top. Data is based on 1000 randomly and uniformly distributed parameter sets that yield normal glucose uptake. The vertical axis is drawn on a linear scale. Note the broad distribution of *S*, which spans multiple orders of magnitude.
(TIF)

S7 Fig Quantitative effects of random parameter changes on glucose uptake. The panel shows the distribution of the absolute value of the relative change in glucose uptake ∣Δ*U*/*U*_*wt*_∣=∣(*U*_*mut*_ − *U*_*wt*_)/*U*_*wt*_∣, where *U*_*wt*_ is the glucose uptake value associated with a randomly sampled viable parameter set, i.e., a set with normal glucose uptake, and *U*_*mut*_ is the glucose uptake value that results if one randomly chosen parameter within this set is randomized, i.e., assigned a new value 10^*x*^, where *x* is a uniform random variate in the interval (−3, 3). The data in the figure is based on 5000 such randomly sampled viable parameter sets. Note the logarithmic scale on the horizontal axis. The data shows that the majority of changes are modest in quantity (*log*_10_∣Δ*U*/*U*_*wt*_∣ ≤ 0).
(TIF)

S8 Fig A parameter’s likelihood *f*_*del*_ to cause deleterious effects when mutated varies broadly across viable parameter sets. Each panel shows the distribution of *f*_*del*_, the fraction of deleterious mutations, for the parameter indicated on top. Note the logarithmic vertical scale. Data is based on 1000 randomly and uniformly distributed parameter sets that yield normal glucose uptake. Note that *f*_*del*_ = 0 for most parameter sets, and that *f*_*del*_ shows a broad distribution.
(TIF)

S9 Fig A parameter’s likelihood *f*_*del*_ to cause deleterious effects when mutated varies broadly across viable parameter sets. For the data in this figure, I first computed fractions of deleterious mutations *f*_*del*_ for all parameters in each of 1000 parameter sets uniformly and randomly sampled from the region of parameter space yielding a normal glucose uptake phenotype. For each parameter set, I then ranked the parameters according to the magnitude of *f*_*del*_. The parameter with the largest *f*_*del*_ received rank one, and all parameters with the smallest possible *f*_*del*_ = 0 received the lowest possible rank of 15. Each panel shows, for the parameter indicated on top, a histogram of the distribution of these ranks among the 1000 parameter sets. Note the logarithmic vertical scale, and that each parameter has the lowest possible rank in the vast majority of the 1000 parameter sets. Nonetheless, each parameter is important (has rank equal or close to one) for a normal glucose-uptake phenotype in some viable parameter sets.
(TIF)

S10 Fig Logistic regression shows that a parameter’s importance for the disease phenotype can vary broadly among populations. Each panel shows a histogram of −*log*_10_ *p* for the parameter indicated on top, where *p* is the *p*-value of the parameter’s regression coefficient in a logistic regression against individuals with normal (‘control’) and impaired (‘case’) glucose uptake. The vertical axis is drawn on a linear scale. Note that the *p*-values vary among many orders of magnitude. Data are based on 100 pairs of populations (parameter sets) that showed either normal or reduced glucose uptake. Each population pair was derived from a single individual with normal glucose uptake and comprised 2000 individuals (See methods for details). Control and case phenotypes are binarily encoded as one and zero, respectively.
(TIF)

S11 Fig Linear regression shows that a parameter’s importance for the disease phenotype can vary broadly among populations. Each panel shows a histogram of −*log*_10_ *p* for the parameter indicated on top, where *p* is the *p*-value of the linear regression coefficient of the parameter against the continuously valued glucose uptake phenotype (equation 15). The vertical axis is drawn on a linear scale. Note that the *p*-values vary among many orders of magnitude. As in the logistic regression analysis, data are based on 100 pairs of populations (parameter sets) that showed either normal or reduced glucose uptake. Each population was derived from a single individual with normal glucose uptake and comprised 1000 individuals (See methods for details).
(TIF)

S12 Fig Linear regression shows that a parameter’s importance for the disease phenotype can vary broadly among populations. Each panel shows a histogram of the rank of *p* for the parameter indicated on top, where *p* is the *p*-value of the linear regression coefficient of the parameter against the glucose uptake phenotype (equation 15). For each parameter set, I ranked parameters according to the magnitude of *p*, such that the parameter with the smallest (most significant) value of *p* received the highest possible rank of one, and that with the largest *p* received the lowest possible rank of 15. The vertical axis is drawn on a linear scale. Note that the ranks have a broad distribution for all parameters, indicating that parameters important in some individuals are unimportant in others. As in the logistic regression analysis, data are based on 100 pairs of populations (parameter sets) that showed either normal or reduced glucose uptake. Each population was derived from a single individual with normal glucose uptake and comprised 1000 individuals (See methods for details).
(TIF)

S13 Fig Additive and multiplicative epistatic interactions explain most of the phenotypic variance. Each panel shows a histogram of the coefficient of determination *R* ^2^ from a regression of the 15 model parameters as predictor variables *x*_*i*_ against the continuous glucose uptake phenotype (equation 15). **a)** linear regression ∑*β*_*i*_ *x*_*i*_; **b)** linear regression with multiplicative interaction terms (∑_*i*_ *β*_*i*_ *x*_*i*_ + ∑_*i* < *j*_ *ε*_*ij*_ *x*_*i*_ *x*_*j*_); **c)** Difference in *R* ^2^ between the multiplicative and the linear model. As in the logistic regression analysis, data are based on 100 pairs of populations (parameter sets) that showed either normal or reduced glucose uptake. Each population was derived from a single individual with normal glucose uptake and comprised 1000 individuals (See methods for details).
(TIF)

S1 Text Supplementary Results. (PDF)

S1 Code Programs to generate data. (ZIP)
