# Supplementary figures and images for: Causal Drift, Robust Signaling, and Complex Disease
Source: PLoS One. 2015 Mar 16;10(3):e0118413. doi: 10.1371/journal.pone.0118413 (PMC4361548; doi:10.1371/journal.pone.0118413)

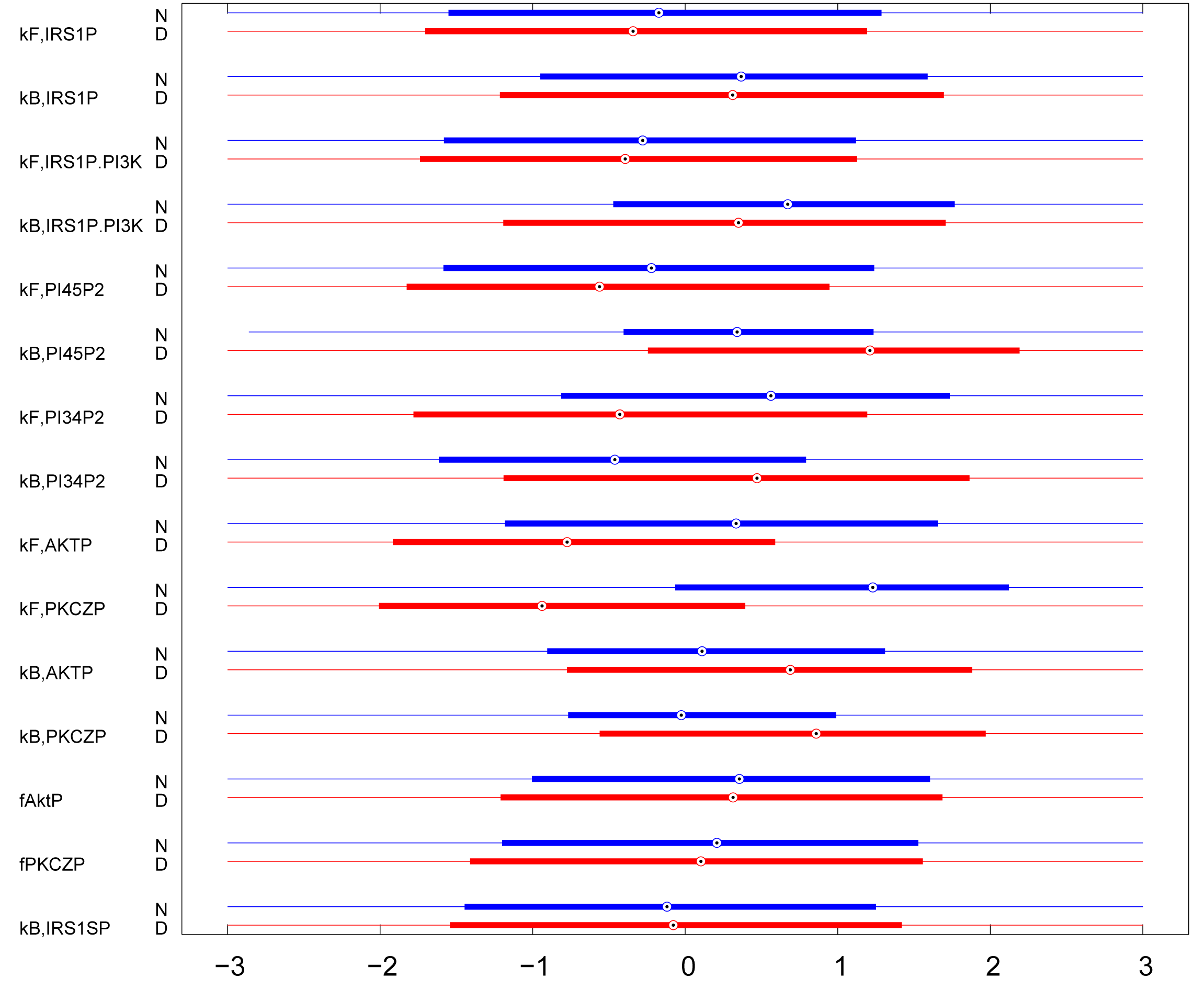

Supplement: S1 Fig — The two box plots next to each parameter name reflect the parameter values that yield normal (‘N’, blue) and reduced (‘D’ for diseased, red) glucose uptake. The horizontal axis (logarithmic scale) covers the admissible parameter range (10−3, 103). The box lot for each parameter is based on 2×10^5^ parameter sets sampled uniformly from a viable region of parameter space (both for normal and reduced glucose uptake). Circles indicate medians, boxes indicate the 25th percentile, q 25, and the 75th percentile, q 75. Whiskers span the interval (q 25 − 1.5(q 75 − q 25), q 75 + 1.5(q 75 − q 25)) corresponding to approximately 99.3 percent coverage of normally distributed data. (TIF) [file pone.0118413.s001.tif]

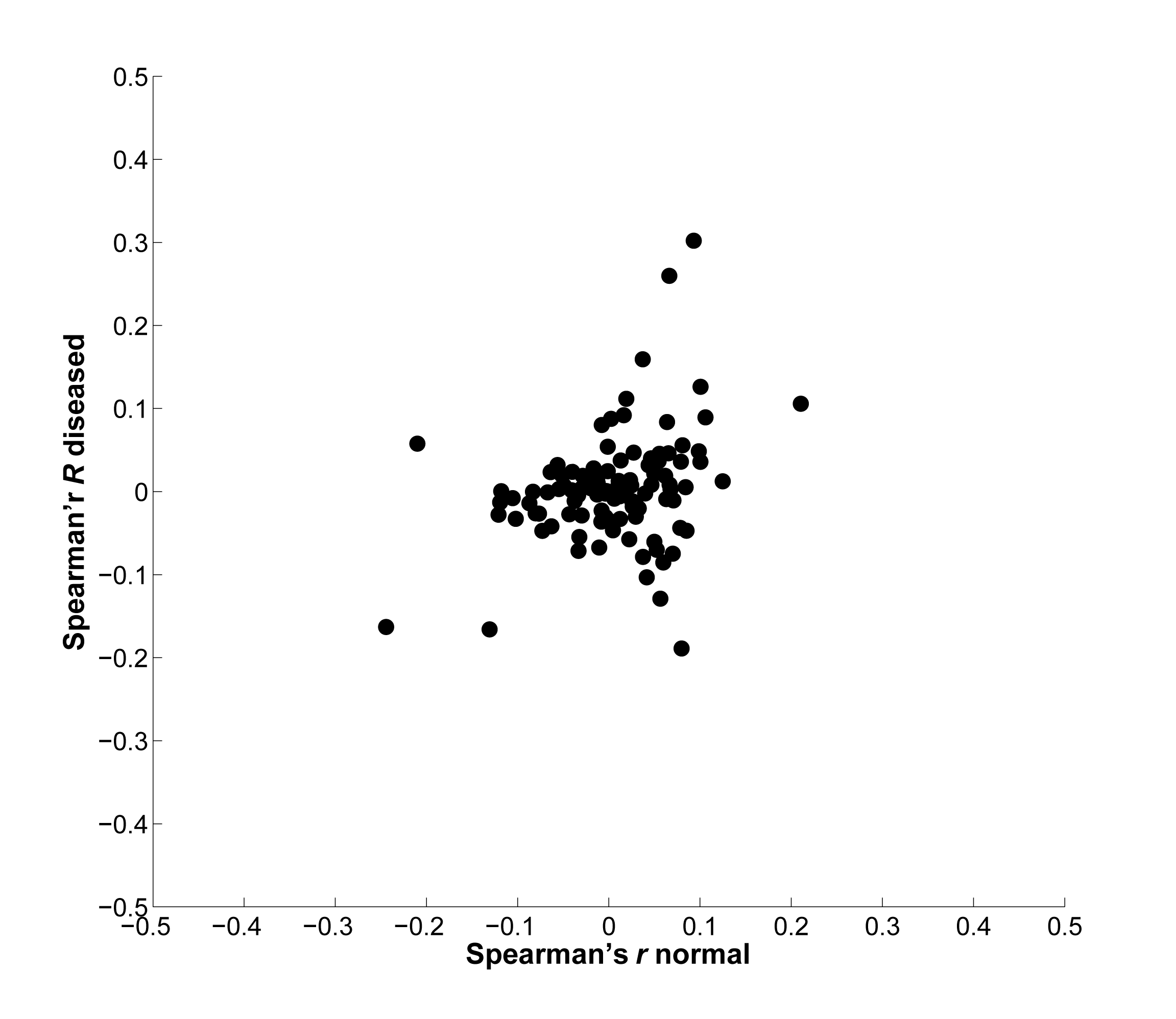

Supplement: S2 Fig — Each data point shows Spearman’s rank correlation coefficient for a pair of parameters, both for normal glucose signaling (horizontal axis) and reduced glucose signaling (vertical axis). Note the axes scales, which extend only to the half-maximal possible values for R. Data are based on 1000 pairs of parameter sets sampled uniformly from the region of parameter space associated with normal or reduced glucose signaling. (TIF) [file pone.0118413.s002.tif]

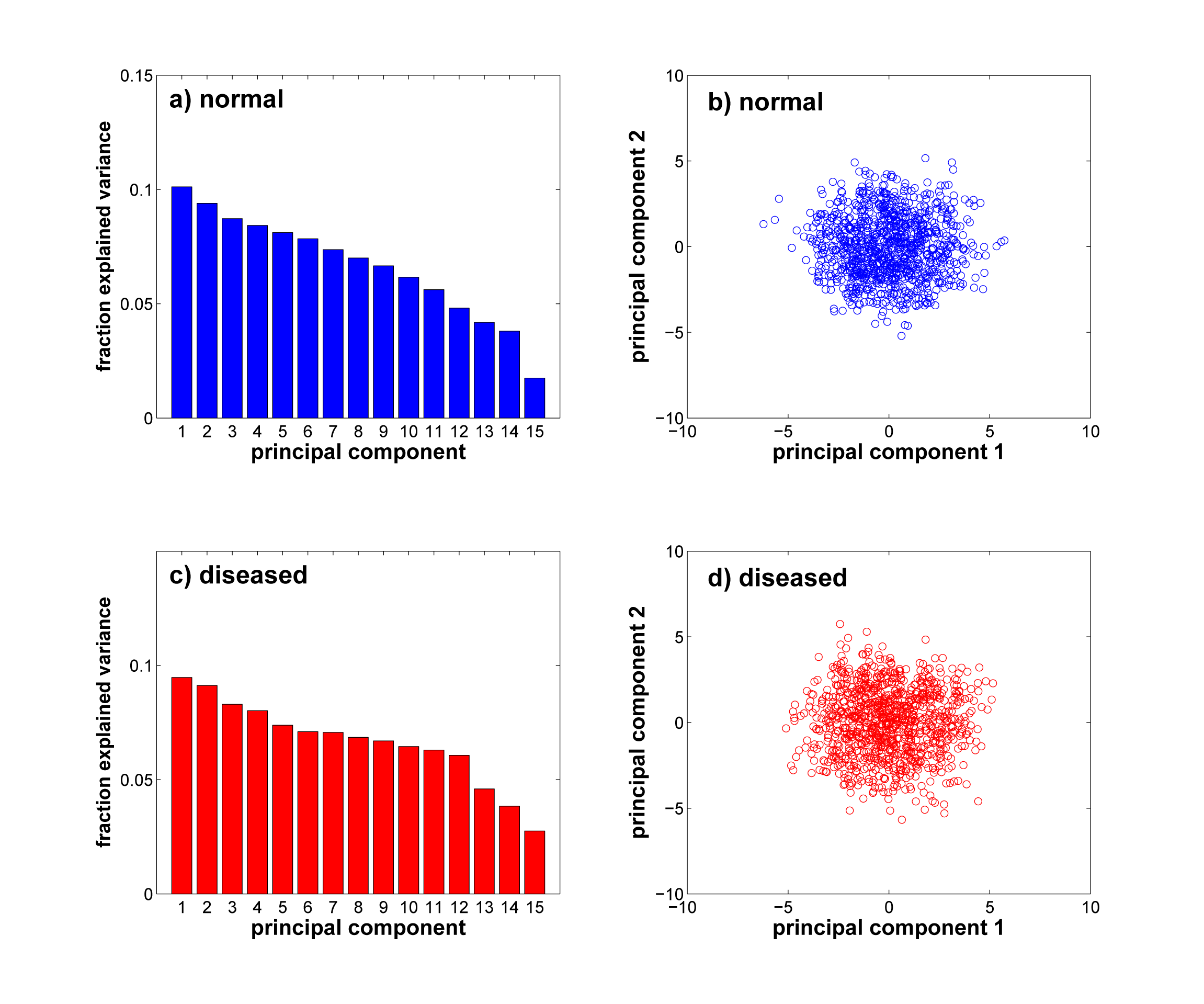

Supplement: S3 Fig — Data are based on 1000 uniformly sampled parameter sets that yield normal (panels a) and b)) or reduced (c) and d)) signaling behavior. Panels a) and c) show the fraction of the variance explained by each of the 15 principal components (horizontal axis) of the 15-dimensional parameter space. Panels b) and d) show the first two principal components plotted against one another. (TIF) [file pone.0118413.s003.tif]

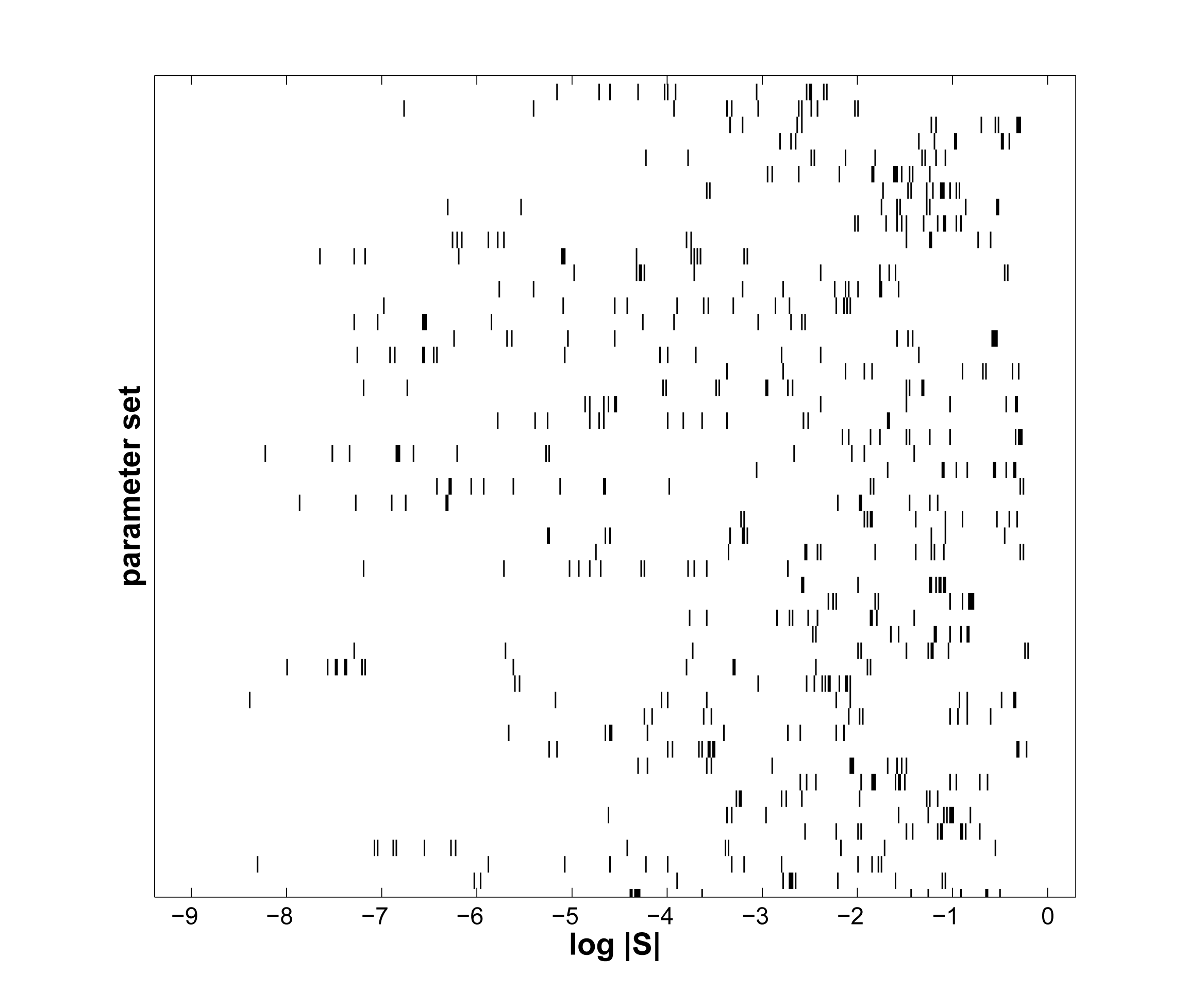

Supplement: S4 Fig — The horizontal axis indicates the log-transformed absolute value of sensitivity coefficients S. The plot has 50 horizontal rows of short vertical bars. Each row corresponds to a different parameter set uniformly and randomly sampled from the viable region of parameter space yielding a normal glucose uptake phenotype. Each row has 15 vertical bars, which indicate the value of S for each of the 15 parameters in the parameter set (For rows where this number of bars appears lower, two or more parameters have values of S that are so similar that some individual bars cannot be resolved). Note that the different parameters have sensitivity coefficients S that vary over multiple orders of magnitude. (TIF) [file pone.0118413.s004.tif]

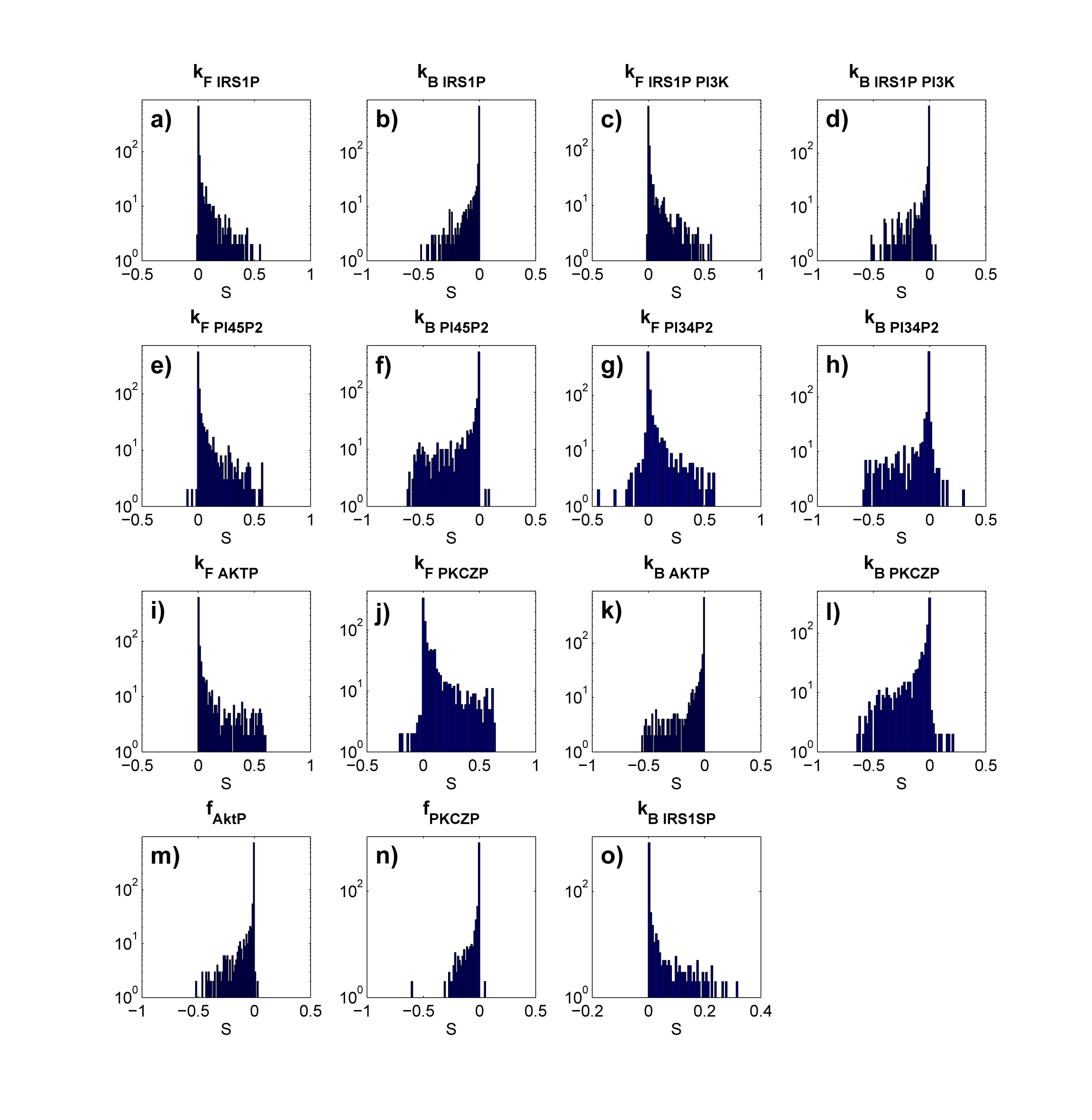

Supplement: S5 Fig — Each panel shows the distribution of sensitivity coefficients for the parameter indicated on top, based on 1000 randomly and uniformly distributed parameter sets that yield a glucose uptake phenotype. Note the logarithmic vertical axis. (TIF) [file pone.0118413.s005.tif]

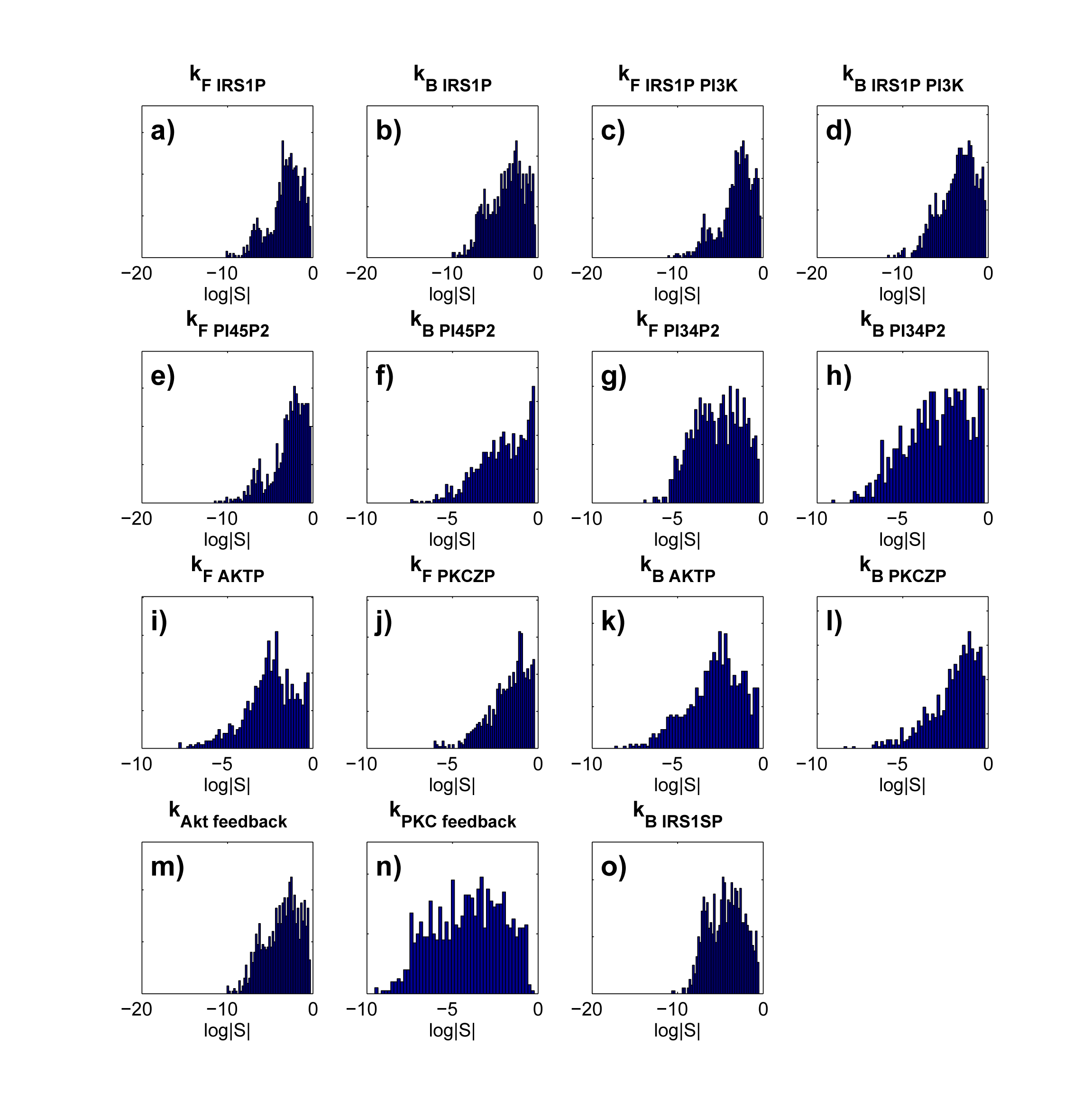

Supplement: S6 Fig — Each panel shows the distribution of log∣S∣, the decadic logarithm of the absolute value of sensitivity coefficients for the parameter indicated on top. Data is based on 1000 randomly and uniformly distributed parameter sets that yield normal glucose uptake. The vertical axis is drawn on a linear scale. Note the broad distribution of S, which spans multiple orders of magnitude. (TIF) [file pone.0118413.s006.tif]

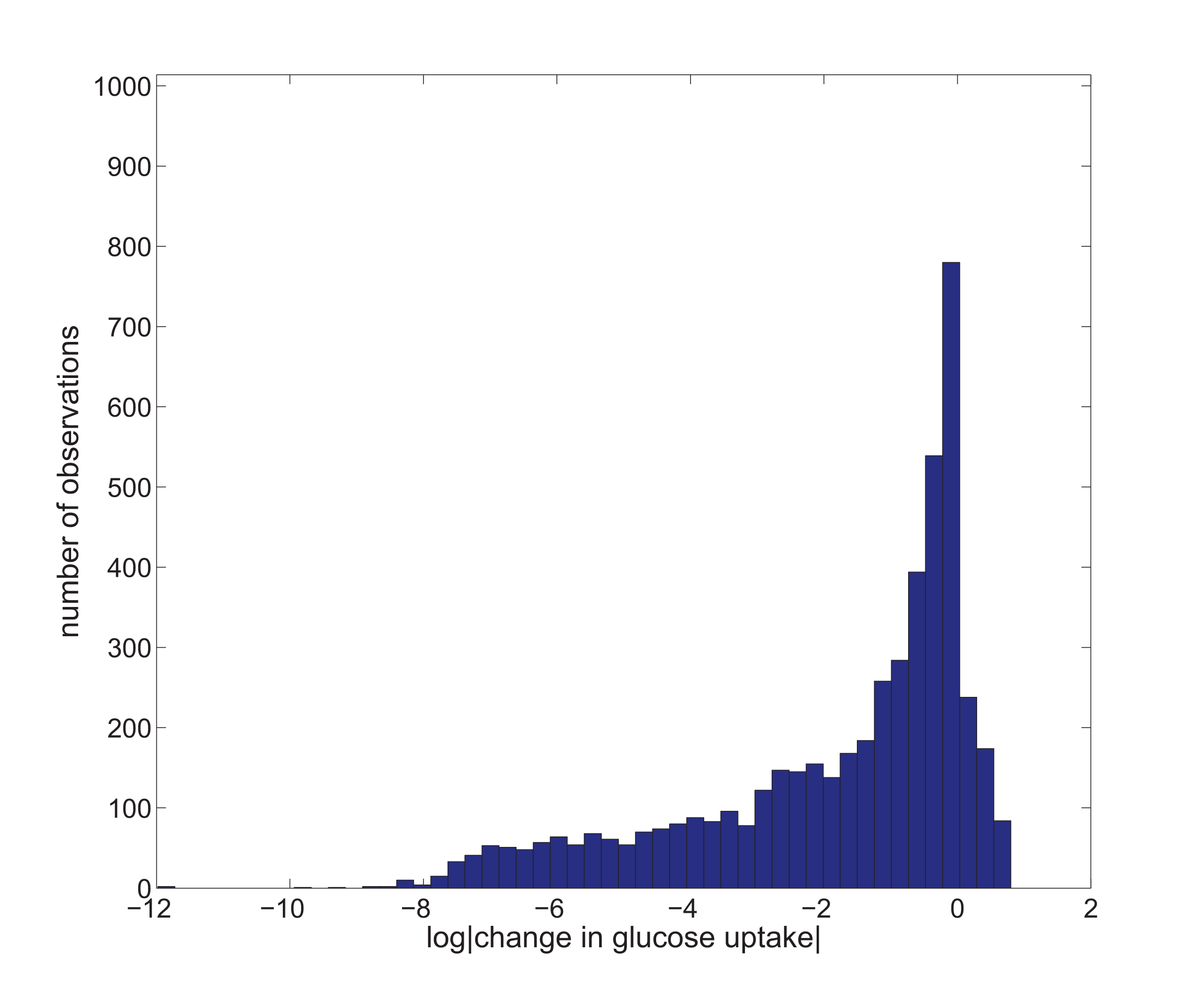

Supplement: S7 Fig — The panel shows the distribution of the absolute value of the relative change in glucose uptake ∣ΔU/U wt∣=∣(U mut − U wt)/U wt∣, where U wt is the glucose uptake value associated with a randomly sampled viable parameter set, i.e., a set with normal glucose uptake, and U mut is the glucose uptake value that results if one randomly chosen parameter within this set is randomized, i.e., assigned a new value 10x, where x is a uniform random variate in the interval (−3, 3). The data in the figure is based on 5000 such randomly sampled viable parameter sets. Note the logarithmic scale on the horizontal axis. The data shows that the majority of changes are modest in quantity (log 10∣ΔU/U wt∣ ≤ 0). (TIF) [file pone.0118413.s007.tif]

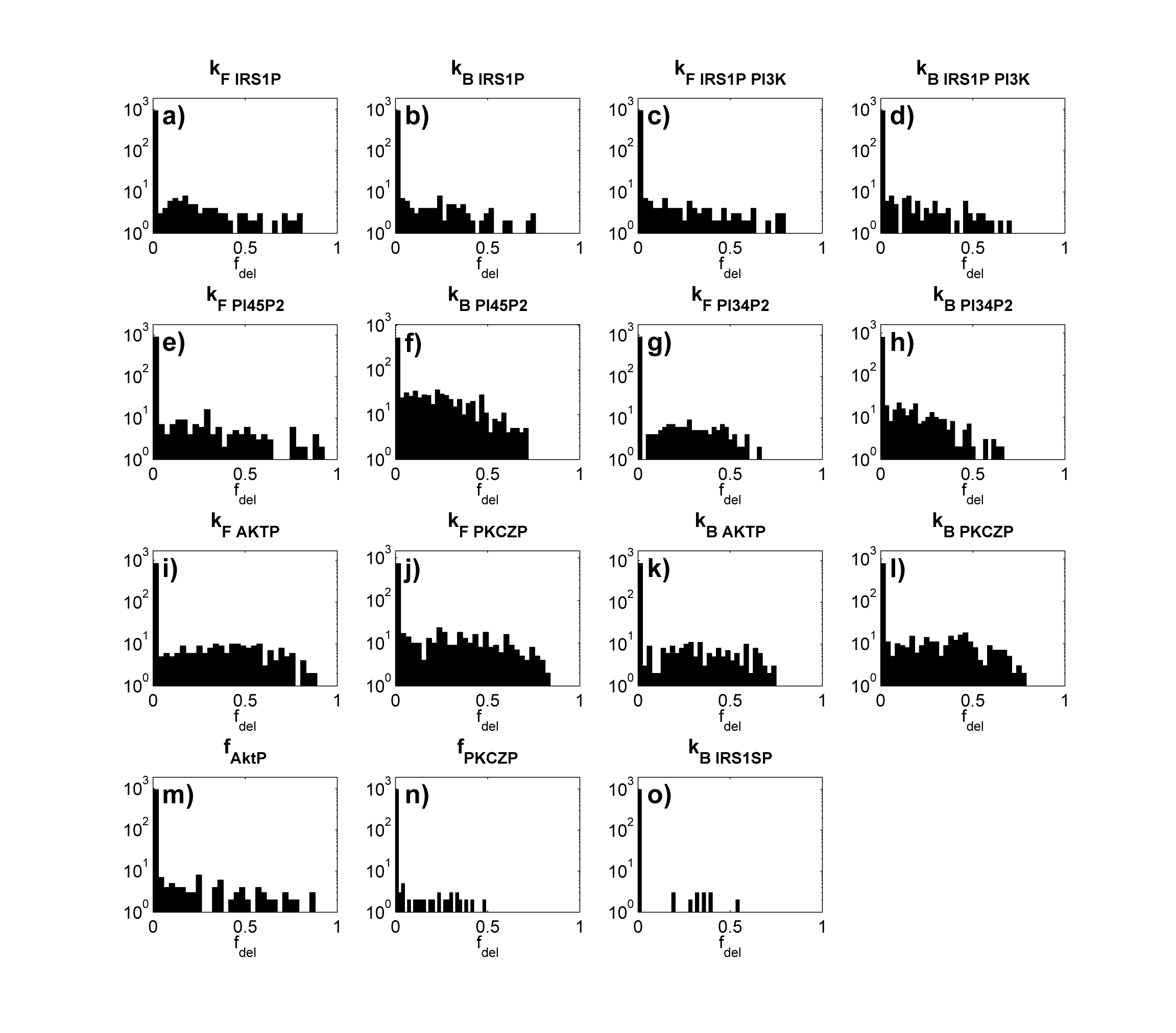

Supplement: S8 Fig — Each panel shows the distribution of f del, the fraction of deleterious mutations, for the parameter indicated on top. Note the logarithmic vertical scale. Data is based on 1000 randomly and uniformly distributed parameter sets that yield normal glucose uptake. Note that f del = 0 for most parameter sets, and that f del shows a broad distribution. (TIF) [file pone.0118413.s008.tif]

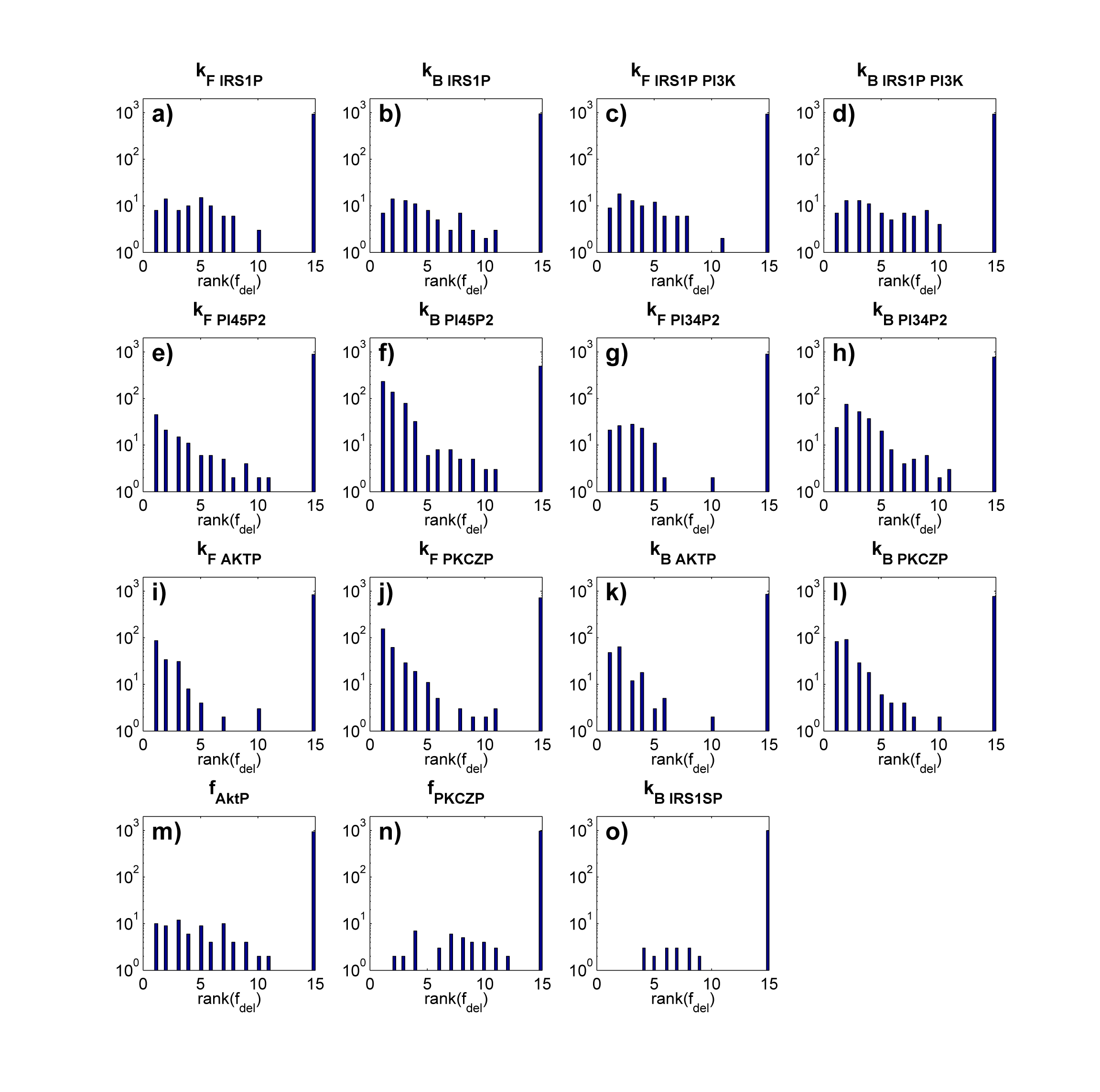

Supplement: S9 Fig — For the data in this figure, I first computed fractions of deleterious mutations f del for all parameters in each of 1000 parameter sets uniformly and randomly sampled from the region of parameter space yielding a normal glucose uptake phenotype. For each parameter set, I then ranked the parameters according to the magnitude of f del. The parameter with the largest f del received rank one, and all parameters with the smallest possible f del = 0 received the lowest possible rank of 15. Each panel shows, for the parameter indicated on top, a histogram of the distribution of these ranks among the 1000 parameter sets. Note the logarithmic vertical scale, and that each parameter has the lowest possible rank in the vast majority of the 1000 parameter sets. Nonetheless, each parameter is important (has rank equal or close to one) for a normal glucose-uptake phenotype in some viable parameter sets. (TIF) [file pone.0118413.s009.tif]

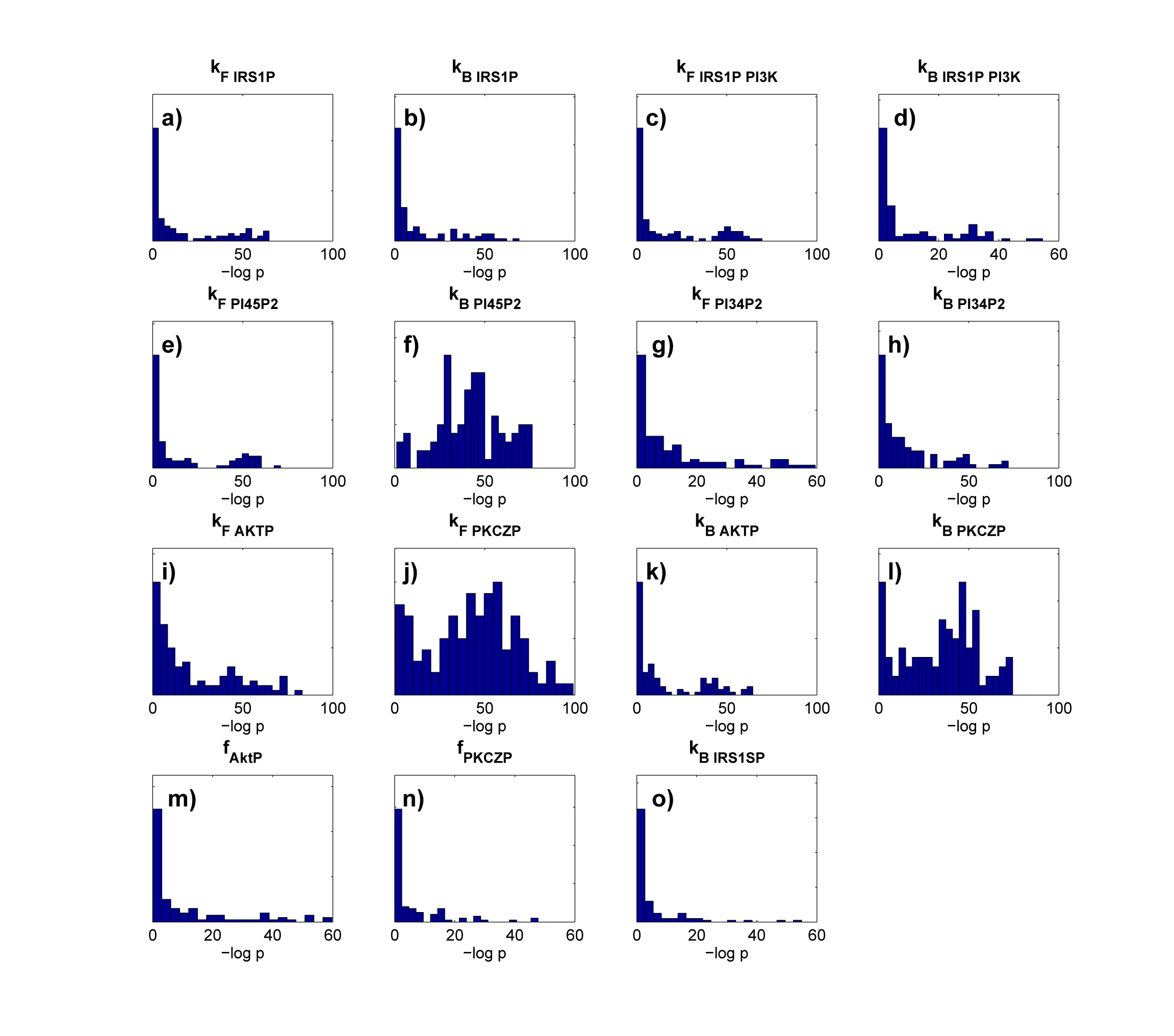

Supplement: S10 Fig — Each panel shows a histogram of −log 10 p for the parameter indicated on top, where p is the p-value of the parameter’s regression coefficient in a logistic regression against individuals with normal (‘control’) and impaired (‘case’) glucose uptake. The vertical axis is drawn on a linear scale. Note that the p-values vary among many orders of magnitude. Data are based on 100 pairs of populations (parameter sets) that showed either normal or reduced glucose uptake. Each population pair was derived from a single individual with normal glucose uptake and comprised 2000 individuals (See methods for details). Control and case phenotypes are binarily encoded as one and zero, respectively. (TIF) [file pone.0118413.s010.tif]

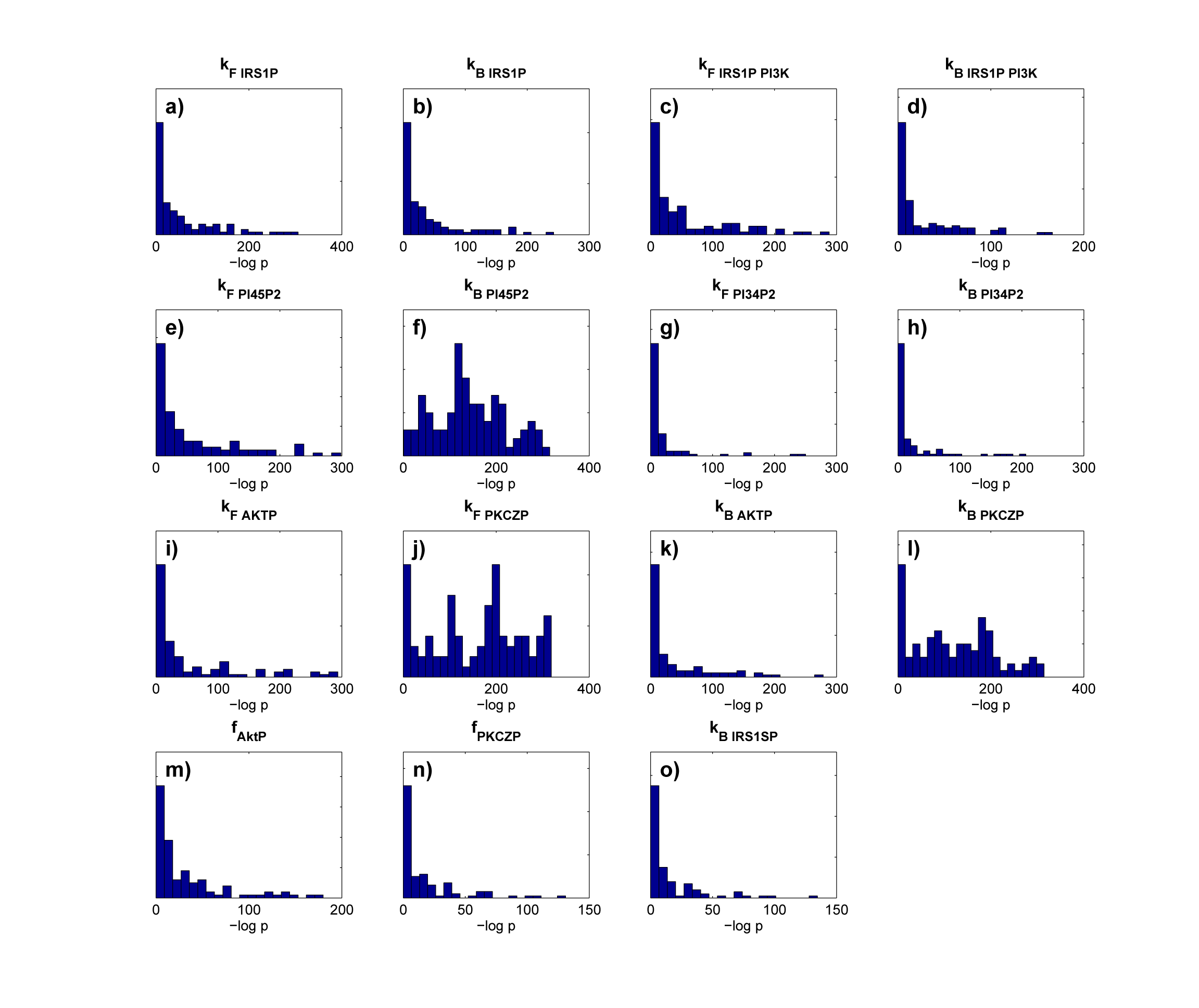

Supplement: S11 Fig — Each panel shows a histogram of −log 10 p for the parameter indicated on top, where p is the p-value of the linear regression coefficient of the parameter against the continuously valued glucose uptake phenotype (equation 15). The vertical axis is drawn on a linear scale. Note that the p-values vary among many orders of magnitude. As in the logistic regression analysis, data are based on 100 pairs of populations (parameter sets) that showed either normal or reduced glucose uptake. Each population was derived from a single individual with normal glucose uptake and comprised 1000 individuals (See methods for details). (TIF) [file pone.0118413.s011.tif]

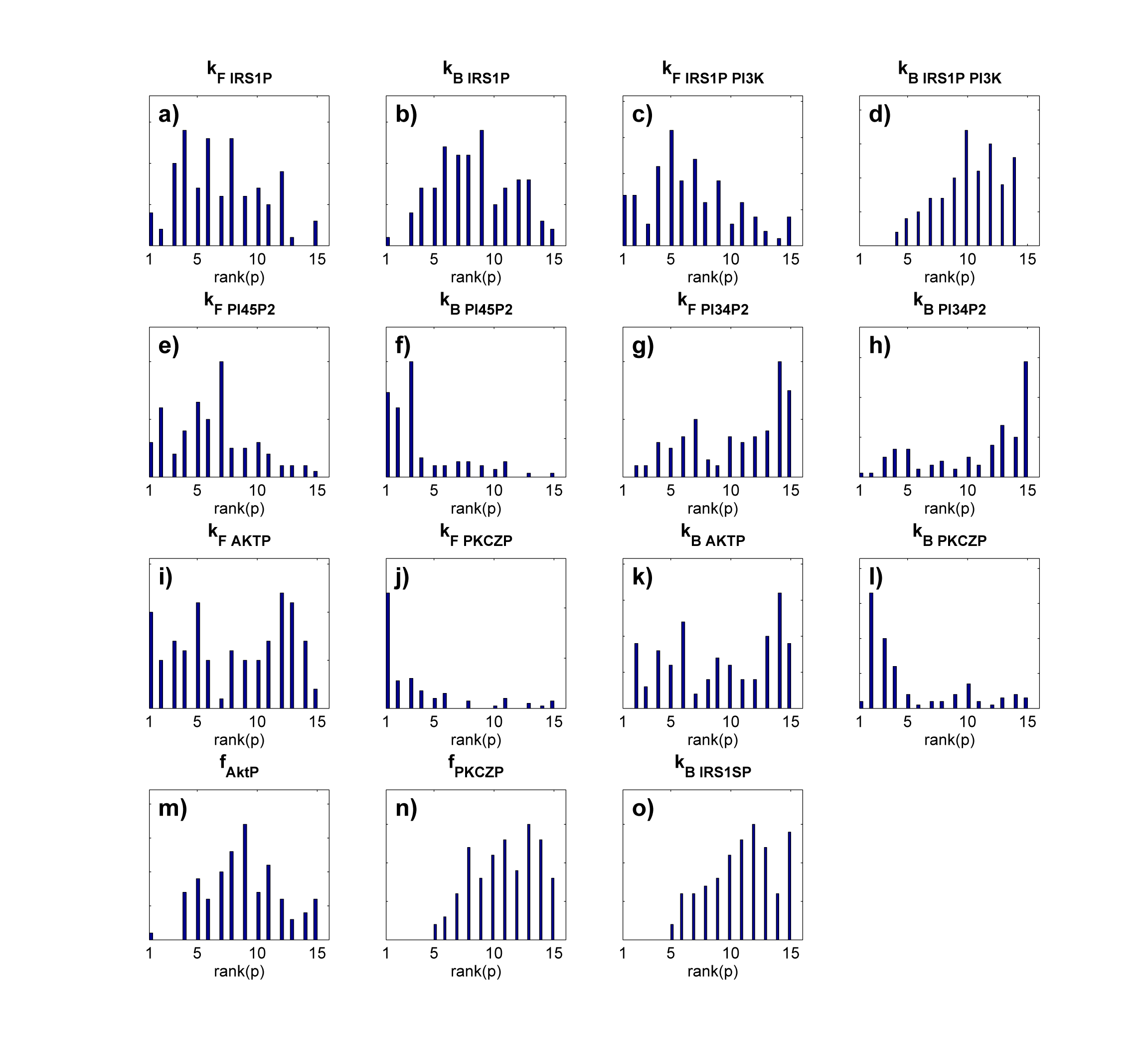

Supplement: S12 Fig — Each panel shows a histogram of the rank of p for the parameter indicated on top, where p is the p-value of the linear regression coefficient of the parameter against the glucose uptake phenotype (equation 15). For each parameter set, I ranked parameters according to the magnitude of p, such that the parameter with the smallest (most significant) value of p received the highest possible rank of one, and that with the largest p received the lowest possible rank of 15. The vertical axis is drawn on a linear scale. Note that the ranks have a broad distribution for all parameters, indicating that parameters important in some individuals are unimportant in others. As in the logistic regression analysis, data are based on 100 pairs of populations (parameter sets) that showed either normal or reduced glucose uptake. Each population was derived from a single individual with normal glucose uptake and comprised 1000 individuals (See methods for details). (TIF) [file pone.0118413.s012.tif]

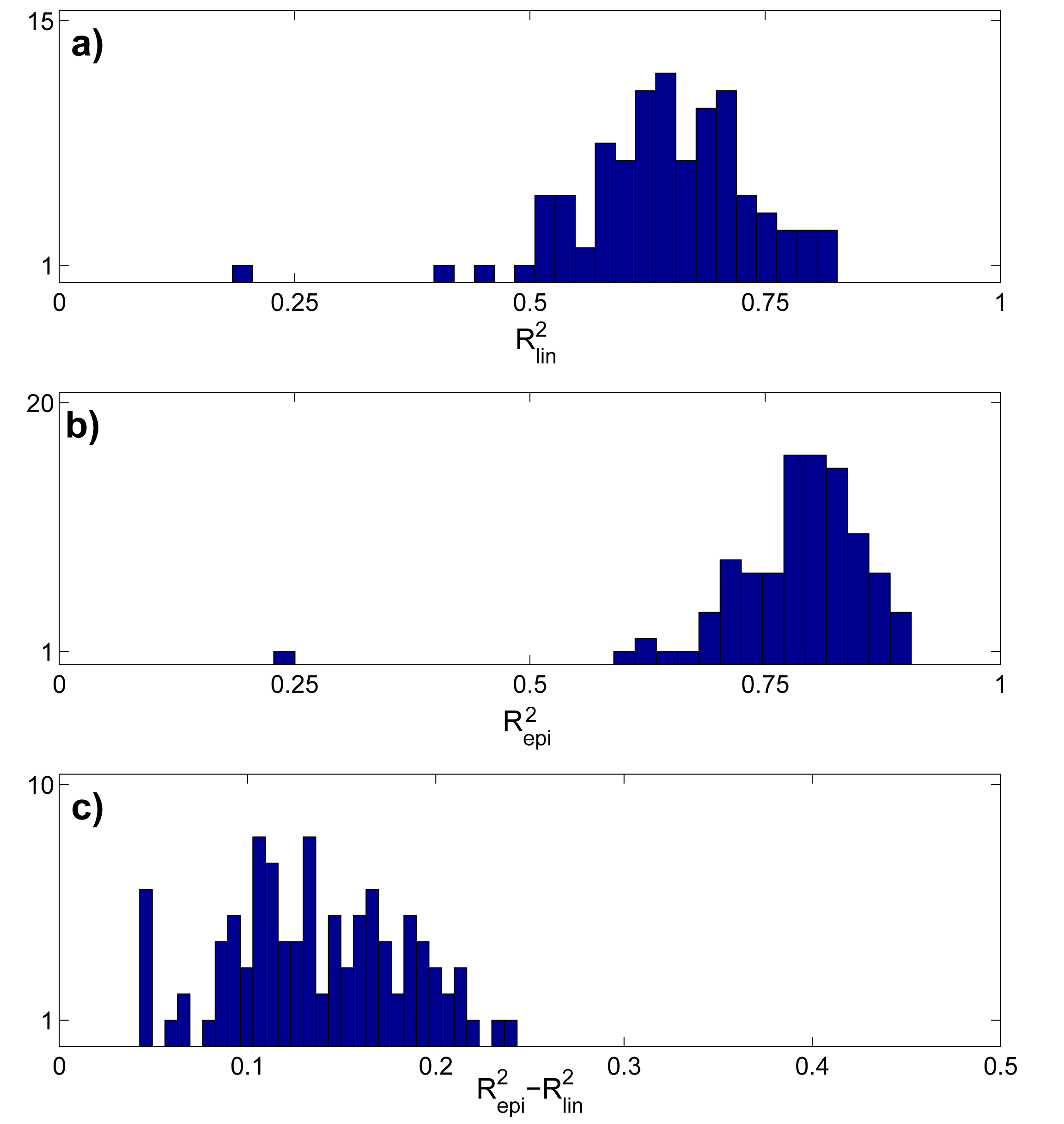

Supplement: S13 Fig — Each panel shows a histogram of the coefficient of determination R 2 from a regression of the 15 model parameters as predictor variables x i against the continuous glucose uptake phenotype (equation 15). a) linear regression ∑β i x i; b) linear regression with multiplicative interaction terms (∑i β i x i + ∑i < j ε ij x i x j); c) Difference in R 2 between the multiplicative and the linear model. As in the logistic regression analysis, data are based on 100 pairs of populations (parameter sets) that showed either normal or reduced glucose uptake. Each population was derived from a single individual with normal glucose uptake and comprised 1000 individuals (See methods for details). (TIF) [file pone.0118413.s013.tif]
