# Supplementary material for: Causal Drift, Robust Signaling, and Complex Disease
Source: PLoS One. 2015 Mar 16;10(3):e0118413. doi: 10.1371/journal.pone.0118413 (PMC4361548; doi:10.1371/journal.pone.0118413)
Supplement: S1 Text — (PDF) [file pone.0118413.s014.pdf]

Causal drift, robust signaling, and complex disease
Andreas Wagner, University of Zurich, andreas.wagner@ieu.uzh.ch

Supplementary Results

Parameter pairs show only weak pairwise associations. I asked whether pairs of parameters are more useful than single parameters in characterizing circuit behavior. For example, if it is crucial that a reaction proceed at an intermediate rate for normal glucose uptake, then increasing values for the parameter determining its forward rate might be accompanied by decreasing values for the parameter determining its reverse rate, leading to a negative association between the two. To find out whether such associations exist, I computed pairwise statistical associations between all pairs of parameters, for uniform samples of 1000 parameter sets that lead to either normal or reduced glucose signaling. Figure S2 shows the Spearman rank correlation coefficients between all possible 105(=$n(n-1)/2$ for $n=15$) parameter pairs. The pairwise associations are quite modest, none of them exceeding $R=0.4$. For circuits with normal signaling behavior, the largest pairwise association exists between $k_{B,PI45P2}$ and $k_{B,PKCZ_P}$, but it is below 0.3 in absolute value ($R=-0.24; P=5.9\times10^{-15}$). In circuits with impaired signaling, the forward and reverse reaction rates $k_{F,PKCZ_P}$ and $k_{B,PKCZ_P}$ show the strongest association, but it also is modest ($R=0.3; P<1.0\times10^{-22}$). In sum, pairwise associations do not help identify circuit components crucial for the signaling phenotype.

Linear combinations of multiple parameters explain a small fraction of viable parameter variation. To find out whether groups of more than two parameters may be more informative, I used principal component analysis, a widely used technique to identify linear combinations of variates (here: individual parameters) that can explain most of the variance in a set of multivariate data (here: viable parameter sets). If any one linear combination of few parameters c_ip_i was able to explain most of the variance in a set of parameters $\{p_i\}$ associated with a specific phenotype (normal or reduced glucose uptake), then these parameters would be especially important determinants of this behavior. Alas, such a parameter combination does not exist. Specifically, the first principal component of the data, which accounts for the largest fraction of the variance, explains only 10.3 percent and 9.9 percent of this variance for parameter sets yielding normal and diseased signaling behavior, respectively (Figure S3). In addition, the proportionality constants c_i (also known as loadings in principal component analysis) are very different for the two samples. In sum, no single linear combination of parameters can encapsulate circuit behavior.

The distribution of ranked f_{del} across parameters shows that parameter importance varies broadly across parameter sets. To assess how strongly the fraction of deleterious mutations f_{del} varies for any one parameter across viable parameter sets, I ranked parameters within any one parameter set according to f_{del}. Specifically, I assigned the lowest possible rank of 15 to a parameter if $f_{del}=0$, and the highest possible

rank of 1 if mutations in the parameter had the largest fraction of deleterious effects among all 15 parameters. Judged by the distribution of these ranks across parameter sets, the importance of parameters varies again broadly (Figure S9). All but two parameters (f_{PKCZP} and $k_{B,IRS1SP}$, Figure S9n and S9o) assume the highest rank in some parameter sets, i.e., their mutation is most likely to cause a deleterious phenotype; they assume intermediate ranks in others, and the lowest rank (never deleterious) in most data sets.

Logistic and linear regression analysis of glucose uptake phenotype. Analogous to logistic regression, I performed 100 different linear regression analyses, one each for 100 pairs of two populations of size 1000, where one population in a pair had normal glucose uptake, and the other had pathologically reduced glucose uptake. I found that p-values for each of the 15 parameters (predictor variables) vary over an enormous range, i.e., between 130 ($k_{B,IRS1SP}$) and 318 ($k_{B,PPKCZP}$) orders of magnitude (Figure S11). And even though all parameters show extremely low (highly significant) p-values in some populations, they are non-significant in others ($p > 0.05$, Figure S11). The fraction of populations where $p > 0.05$ ranges from 1 percent ($k_{B,PI45P2}$) to 26 percent ($k_{B,PI34P2}$), with a mean of 12.9 percent. When ranking each of the 15 parameters according to its p-value for a population, I find that the ranks vary broadly, i.e., they have the maximally possible range (1-15) for 10 parameters, and only somewhat lower ranges for the others ($k_{B,IRS1P.PI3K}$: 4-14; $k_{F,PI34P2}$: 2-15; $k_{B,AKTP}$: 2-15; f_{PKCZP}; and $k_{B,IRS1SP}$: 5-15; see Figure S12). In sum, a parameter important to explain changes in glucose uptake in one population typically has greatly diminished importance in some other population.

Linear regression also allows one to compute the fraction of a population's phenotypic variance that can be explained through the sum of the predictor variables x_i ($\sum \beta_i x_i$, where the β_i's are regression coefficients) via the coefficient of determination R^2_{lin}. The distribution of R^2_{lin} (Figure S13a) shows that in most populations, most phenotypic variation can be explained additively. Although one might naively conclude from this observation that an additive model would be useful to explain phenotypic variation, the extreme variability of parameter importance shows that this interpretation would be misleading. The relative importance of predictor variables (parameters) in a truly linear model would be essentially the same in different populations of the size I study, but this is emphatically not the case in the insulin signaling circuit, where a parameter may have a high importance in some populations and low importance in others.

A recurrent question in genetic association studies regards the prevalence of epistatic (non-additive) interactions among pairs of loci. In the insulin signaling circuit there are sufficiently few such interactions (among parameters) to study the contribution of all pairwise interactions, and ask whether they are important to predict the phenotype. To this end, I performed a regression analysis that aims to predict phenotypic variance through the expression $\sum_i \beta_i x_i + \sum_{i<j} \epsilon_{ij} x_i x_j$, i.e., it allows multiplicative interactions among all pairs of parameters. For each of the 100 populations, I computed the fraction of variance R^2_{epi} explained by this epistatic model (Figure S13b). I found that R^2 ranges between 0.23 and 0.91, depending on the population). By subtracting the linear coefficient

of determinationR^2_{lin} from it one obtains the fraction of variance explained only by multiplicative interactions $R^2_{epi} - R^2_{lin}$. This fraction is generally small (Figure S13c), explaining between 4.3 and 24.3 percent of the variance, depending on the population. The remaining variance would have to be explained by non-multiplicative pairwise epistasis or by higher order interactions. And even though this fraction of remaining variance is usually not high (Figure S13b), the same caution applies as to linear regression: The high fraction of explained variance can create the illusion that the contribution of individual disease determinants to phenotype is well-captured by a simple additive-multiplicative model, where this contribution would not vary greatly among populations.

References

1. Joliffe I (2002) Principal component analysis. New York, NY: Springer, 2nd edition.
2. Sokal R, Rohlf F (1981) Biometry. New York: Freeman.
